# Ecological and Historical Correlates of Taxonomic, Phylogenetic, and Functional Diversity of Amphibians in South American Rainforests and Savannas

**DOI:** 10.1002/ece3.73494

**Published:** 2026-04-16

**Authors:** Thiago Gonçalves‐Souza, Lilian S. O. Melo, Ana C. Carnaval, Denise de C. Rossa‐Feres, Andrea Paz, Ivan Prates

**Affiliations:** ^1^ Institute for Global Change Biology, School for Environment and Sustainability University of Michigan Ann Arbor Michigan USA; ^2^ Department of Ecology and Evolutionary Biology University of Michigan Ann Arbor Michigan USA; ^3^ Programa de Pós‐Graduação em Biologia Animal, Departamento de Ciências Biológicas Universidade Estadual Paulista Júlio de Mesquita Filho, UNESP São José do Rio Preto São Paulo Brazil; ^4^ Department of Biology, City College of New York, and Graduate Center City University of New York New York New York USA; ^5^ Département de Sciences Biologiques Université de Montréal Québec Canada; ^6^ Department of Biology Lund University Lund Sweden

**Keywords:** Amazonia, Atlantic Rainforest, Cerrado, functional diversity, phylogenetic diversity, taxonomic diversity

## Abstract

We investigate the ecological and evolutionary variables that best explain spatial diversity patterns of anuran amphibians in three of South America's most diverse and geographically widespread biomes: the Cerrado, Amazonia, and Atlantic Rainforest. We used Conditional Autoregressive Models to assess the potential influence of present‐day climate (temperature and precipitation), historical climate (stability over the last 120,000 years), potential evapotranspiration (PET), and topography (slope, aspect, and rugosity) on spatial variation in taxonomic, functional, and phylogenetic diversity at a resolution of 0.5 × 0.5 degrees. Both taxonomic diversity and phylogenetic diversity increased with long‐term climatic stability in all regions. By contrast, functional diversity was negatively impacted by precipitation in the driest quarter. However, the relative importance of each predictor variable differed among diversity metrics and biomes. In the Atlantic Rainforest, potential evapotranspiration was positively correlated with functional diversity but negatively associated with taxonomic and phylogenetic diversity. In Amazonia, precipitation and relief slopes were positively associated with functional and phylogenetic diversity, respectively, whereas relief slopes were negatively correlated with taxonomic diversity. In the Cerrado, precipitation was negatively correlated with functional diversity, but climatic stability was more strongly associated with phylogenetic and taxonomic diversity. These findings indicate that present‐day climatic factors are critical in forested biomes, whereas a combination of historical and current variables is more relevant in Cerrado's savanna mosaics. Notably, ecotones exhibit significantly higher functional diversity (except for Amazon), reflecting the encounter of faunas from adjacent biomes having distinct ecological regimes and thus associated organismal traits. Characterizing the drivers of heterogeneous biodiversity distribution will offer insights into the assembly of ecological communities in other tropical and transitional ecosystems and can potentially guide conservation strategies globally.

## Graphical abstract

This study identifies the key drivers of anuran amphibian diversity (taxonomic, functional, and phylogenetic) across South America's Cerrado, Amazonia, and Atlantic Rainforest. Although taxonomic and phylogenetic diversity consistently increased with long‐term climatic stability in all regions, functional diversity was primarily influenced by current precipitation and showed divergent regional responses to predictors like potential evapotranspiration and topography. The findings reveal distinct combinations of historical and contemporary factors shaping each diversity dimension per domain, with notably higher functional diversity in ecotones where adjacent faunas meet.

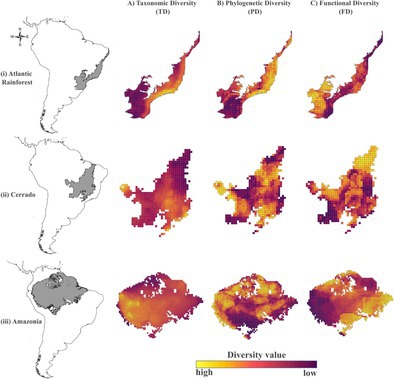

## 1 Introduction

Identifying the drivers of species richness and turnover is crucial to explaining why life on Earth is heterogeneously distributed. Macroecological studies have found that spatial environmental gradients can predict continental‐scale biodiversity patterns across many groups of organisms and geographic regions (Prieto‐Torres et al. 2020; Sales et al. 2020). Beyond species (i.e., taxonomic) diversity, these approaches have increasingly sampled additional biodiversity metrics, such as functional and phylogenetic diversity (Hidasi‐Neto et al. 2013; Hähn et al. 2025). Distinct biodiversity metrics often co‐vary in response to the same environmental factors. For example, temperature and precipitation variation predict taxonomic and functional diversity concurrently in certain plant clades (Cadotte et al. 2011). However, biodiversity metrics can also respond differently to environmental variables (de Pauw et al. 2021). These contrasting patterns suggest context‐dependency in the factors underlying community assembly, with varying contributions of distinct ecological and historical variables across geographic regions (Rodrigues‐Filho et al. 2017; de Pauw et al. 2021; Hähn et al. 2025).

In the biologically diverse and threatened Neotropical region, we have a limited understanding of how environmental gradients contribute to the accumulation of taxonomic, lineage, and trait diversity. Even among habitats considered similar, distinct environmental descriptors appear to explain regional biodiversity (Qian et al. 2017). For instance, da Silva et al. (2012) found that the richness of regional frog species pools in the Brazilian Atlantic Rainforest increases with water availability, which also predicts higher phylogenetic and reproductive trait diversity. Conversely, Wiens et al. (2011) found that climate variables do not predict variation in frog richness at some sites in Amazonia. Such conflicting patterns between the Atlantic Rainforest and Amazonia—both representing the tropical rainforest biome—suggest that the primary drivers of biodiversity may be region‐specific (e.g., Moncrieff et al. 2015, 2016). However, drawing general patterns across regions is challenging because studies have typically focused on a single biome while differing markedly in methodology, spatial scale, and phylogenetic scope (Wiens et al. 2011; Neves et al. 2017; Paz et al. 2021).

To compare correlates of biodiversity patterns across Neotropical regions and biodiversity metrics, we map taxonomic, phylogenetic, and functional diversity within a common analytical framework. We focus on amphibians, a group with strong habitat associations (Smith and Green 2006; Gamble et al. 2007), limited dispersal ability (Smith and Green 2005), and high sensitivity to environmental shifts (Beebee and Griffiths 2005), as illustrated by human‐driven declines worldwide (Grant et al. 2016; Rebouças et al. 2021). Given these aspects of amphibian biology, we expect spatial environmental gradients to strongly affect regional amphibian diversity. Additionally, we anticipate that climate and habitat history contribute to patterns of taxonomic and phylogenetic diversity. For example, regional climatic stability during the Pleistocene might have favored frog species accumulation through long‐term population persistence (Martínez‐Monzón et al. 2021). Emerging topographic barriers might favor population isolation and divergence, thereby increasing taxonomic (Rahbek et al. 2006), phylogenetic (Mosbrugger et al. 2018), and functional diversity (Monge‐González et al. 2021). Conversely, long‐standing environmental regimes might favor convergent trait evolution across species, leading to decreased relative functional diversity (Lamanna et al. 2014). Previous analyses have found that present‐day climatic gradients contribute to amphibian taxonomic turnover within the Atlantic Forest and Cerrado (Luiz et al. 2016; Valdujo et al. 2013), while being less important in Amazonia (Godinho and da Silva 2018). Meanwhile, a single analysis focused on the Atlantic Forest has incorporated historical climatic stability, finding it to explain part of amphibian taxonomic and phylogenetic turnover (Da Silva et al. 2014). However, we still lack a broader understanding of the relative importance of present‐day and historical factors on taxonomic, phylogenetic, and functional biodiversity patterns across regions.

To explore common versus region‐specific correlates of biodiversity patterns in the Neotropics, we focused on three biogeographic biomes that vary in present‐day climate, topographic complexity, and long‐term environmental stability: Amazonia, the Atlantic Rainforest, and the Cerrado. Despite hosting exceptionally high numbers of species, these regions have rarely been compared under the same framework, limiting comparisons of the relative role of environmental factors in the composition of regional communities. We expect such factors to impact each region differently. For instance, variation in topography and temperature might more strongly affect amphibian diversity in the Atlantic Rainforest and Amazonia, which span both lowlands and montane areas (see, e.g., Jiménez‐Robles et al. 2017). In turn, long‐term climatic stability might be particularly important in the Atlantic Rainforest and Cerrado, given their dynamic history of climatic shifts during the Quaternary relative to the more climatically stable Amazonia (Baker et al. 2020). In turn, species diversity in all three biomes should increase with temperature, precipitation, topographic complexity, potential evapotranspiration, and historical climatic stability. We seek to address three major questions. First, what are the most important environmental correlates of regional variation in amphibian taxonomic, phylogenetic, and functional diversity? Second, do these drivers affect biodiversity differently in each region? Third, are these diversity metrics coupled or decoupled across regions?

## 2 Materials and Methods

### 2.1 Biome Overview

Amazonia is the largest rainforest biome of the world, spanning ca. 7,000,000 km^2^ across nine South American countries. Most (ca. 90%) of its extension experiences a humid tropical climate (Peel et al. 2007) with a mean annual precipitation of 2130 mm (Costa and Foley 1998) and average temperatures from 22°C to 26°C (Alvares et al. 2013). Humid tropical forests predominate, but the biome also encompasses floodplain forests and savannas (Arruda et al. 2018). Amazonia's current configuration dates back to the Late Miocene, after the onset of Andean tectonism and establishment of major Amazon basin rivers (Hoorn et al. 1995, 2010; Figueiredo et al. 2009).

The Cerrado of central Brazil is South America's second largest biome, comprising more than 2,000,000 km^2^ (Klink and Machado 2005). It encompasses a mosaic of dry forest, deciduous forest, and dense, open, and flooded savannas (Silva et al. 2006), which host a high number of species (Valdujo et al. 2012). The Cerrado has a tropical climate with a pronounced dry season (Peel et al. 2007), annual precipitation from 800 to 2000 mm, and average temperatures from 18°C to 28°C (Dias 1992). Natural fire dynamics dictate local vegetation types (Segura‐Garcia et al. 2025). The major events in Cerrado's geological history are the formation of vast plateaus (from 500 to 1700 m) in the Late Tertiary (6 mya) and valleys (100–500 m) in the Early Quaternary (1.6 mya) (Ab'Saber 1983; Silva 1997).

Finally, the Atlantic Rainforest comprises 1,500,000 km^2^ along a pronounced latitudinal gradient (7.4° S–34.8° S) from northeastern to southern Brazil (Ministério do Meio Ambiente (MMA) 2017), harboring diverse vegetation types (e.g., coastal, dense, mixed, and semi‐deciduous forests; Oliveira‐Filho and Fontes 2000). Climate is mostly tropical humid, turning to temperate in the south (Peel et al. 2007). Precipitation and temperature vary widely across the biome, with annual rainfall from 1300 to 2500 mm and average temperature from 12°C to 26°C (Alvares et al. 2013). The Atlantic Rainforest encompasses mountain chains such as the Serra do Mar and Mantiqueira, whose formation started in the Paleocene (65 mya) with major uplifts in the Miocene (23 mya) (Almeida and Carneiro 1998).

In our analyses, biome boundaries followed the Brazilian Ministry of Environment for the Atlantic Rainforest and Cerrado (Ministério do Meio Ambiente (MMA) 2017) and the World Wildlife Fund for Amazonia (Olson et al. 2001); the Atlantic Rainforest boundaries defined by Ministério do Meio Ambiente (MMA) (2017) include only the Brazilian biome. In Amazonia, we restricted our analyses to the evergreen broadleaf forest to the east of the Andes, masking out the *babaçu* and seasonally dry forests.

### 2.2 Species Ranges and Grid Cell Species Composition

We used IUCN expert range maps of 1096 taxa from the IUCN Global Amphibian Assessment (IUCN 2023) to obtain the list of amphibian species in each grid cell under the same spatial resolution as the environmental data (0.5° × 0.5° grid cell). To obtain maps of taxonomic composition per pixel, we extracted species lists for each cell from superimposed range maps using the R package letsR (Vilela and Villalobos 2015). We controlled for taxonomic synonymy on the basis of the Amphibians of the World Database (Frost 2018). Five species were excluded from downstream analyses owing to uncertain taxonomic status ( *Hyloxalus peruvianus* , *Ischnocnema bilineata* , *Rhinella pombali* , and *Tepuihyla warreni* ) or recent extinction ( *Phrynomedusa fimbriata* ). We acknowledge the limitations of predicting species richness and composition from IUCN range maps (Herkt et al. 2017); however, multiple studies indicate that these maps provide reasonable approximations for macroecological analyses of vertebrates (Alhajeri and Fourcade 2019; Aronsson et al. 2024; Broekman et al. 2022).

### 2.3 Phylogenetic Diversity

We estimated phylogenetic diversity at each cell on the basis of the consensus phylogeny of Jetz and Pyron (2018), which incorporated GenBank DNA sequences to generate a comprehensive amphibian phylogeny. To tentatively place in the tree taxa with no genetic data available, Jetz and Pyron (2018) used Phylogenetic Assembly with Soft Taxonomic InferenceS (PASTIS) on the basis of taxonomic assignments (Jetz et al. 2012; Thomas et al. 2013). We placed 15 species not present in Jetz and Pyron's phylogeny as polytomies within the corresponding family or genus‐level clade.

On the basis of this tree, we estimated phylogenetic diversity in each grid cell using the net relatedness index (NRI) (Webb et al. 2002). NRI is a standardized measure of the mean pairwise phylogenetic distance between taxa in a grid relative to a phylogeny that includes the regional species pool. This pool was defined as all species present in the three biomes. Phylogenetic distances corresponded to the total branch lengths separating two taxa. By corresponding to a standardized effect size of mean pairwise distances (MPD) (Webb et al. 2002), the estimation of phylogenetic diversity on the basis of NRI is independent of the number of species; this property is essential because we want to understand the environmental correlates determining regional diversity in terms of evolutionary history, and therefore calculating an independent metric is relevant to tease apart potential differences among metrics. Hereafter, we refer to NRI simply as phylogenetic diversity (PD).

### 2.4 Trait Collection, Imputation, and Functional Diversity

We compiled functional traits on the basis of the scientific literature (especially taxonomic descriptions), books, and expert knowledge (Table S2). We considered three quantitative and two qualitative morphological and developmental traits related to dispersal capacity (tibia length), feeding (head width), size (snout‐vent length), development (direct or indirect), and tadpole biology, in three nominal categories: endotrophic, that is, non‐feeding larvae that can be (i) non‐hatched or (ii) free‐swimming, or (iii) exotrophic, that is, feeding, free‐swimming larvae. Details on the ecological relevance of each selected trait are provided in Table S3.

We obtained trait data for most species, but 180 species (16.5% of all species) had missing information for some quantitative trait. To perform trait imputation and simulate trait values for those species with missing data, we used Phylopars (Bruggeman et al. 2009) (Supporting Information: Material S3, Table S4). We tested whether data imputation changed observed patterns of trait evolution by confirming the presence of phylogenetic signal in both the imputed and non‐imputed datasets on the basis of the K statistic (Blomberg et al. 2003). In the case of categorical trait data, we tested for phylogenetic signal using the method from Maddison and Slatkin (1991), where the tree is fixed, and traits are shuffled randomly. Additional details about imputation and phylogenetic signal methods and results are available in Supporting Information: Material S3 and Tables S4 and S5.

To calculate functional diversity on the basis of the compiled traits, we used a method that accounts for the phylogenetic relatedness between species (de Bello et al. 2017). We first created a functional pairwise dissimilarity matrix using the Gower index (Pavoine et al. 2009), which combines quantitative and nominal trait values. To assess species relatedness, we used a Principal Coordinate Analysis (PCoA) on the phylogenetic pairwise dissimilarity matrix to extract phylogenetic eigenvectors (Diniz‐Filho et al. 2012). Then, we regressed these phylogenetic eigenvectors against the functional dissimilarity matrix using a Redundancy Analysis (RDA). The residuals of the fitted regression correspond to the variation in species' traits that is independent of the phylogeny (de Bello et al. 2017). Then, we calculated functional diversity per grid cell on the basis of the functional dispersion index (Laliberté and Legendre 2010) using the residuals of the previous regression analysis. This index corresponds to the average distance from the centroid of a volume defined by the traits (after accounting for phylogenetic relatedness). We applied a square root transformation on the adjusted trait data, owing to negative eigenvalues that often result from the PCoA procedure (Legendre and Anderson 1999). The estimated functional diversity has the advantage of not being affected by the number of species and being robust to outliers (Anderson 2006; Laliberté and Legendre 2010).

### 2.5 Present‐Day Environmental Variables

To explore which environmental descriptors best explain spatial patterns of amphibian diversity, we obtained present‐day and Pleistocene temperature and precipitation data from the Hadley Center Climate models at a 0.5° × 0.5° resolution (Singarayer and Valdes 2010; Fuchs et al. 2013). For historical climates, we considered the past 120 kyr with a time interval of 4 kyr. We used Hadley Center simulations because they provide temperature and precipitation reconstructions across the Late Quaternary. The 4 kyr time interval was chosen because paleoclimatic reconstructions for older periods are only consistently available at this temporal resolution, requiring standardization across all time slices. We selected the following bioclimatic variables because they are frequently considered in studies of both amphibians and Neotropical forests and savannas: annual mean temperature (Bio 01), mean temperature of the warmest quarter (Bio 10), mean temperature of the coldest quarter (Bio 11), annual precipitation (Bio 12), precipitation of the wettest quarter (Bio 16), and precipitation of the driest quarter (Bio 17). Additionally, we extracted potential evapotranspiration of the warmest quarter (PET) data from the ENVIREM database (Title and Bemmels 2018) at a 0.5° × 0.5° resolution. We included PET as a measure of atmospheric water demand, which is related to desiccation risk and energetic stress in amphibians (Olalla‐Tárraga et al. 2009). We decided to use 0.5° resolution because it is commonly used in continental‐scale macroecological analyses (Olalla‐Tárraga et al. 2009; Biber et al. 2020).

We extracted elevation data from the Shuttle Radar Topography Mission (Jarvis et al. 2008) and calculated slope, aspect, and roughness surfaces on a 0.5° × 0.5° resolution using the R package raster (Hijmans, van Etten, et al. 2025). These metrics of topographic variation were shown to predict regional and continental‐scale species diversity patterns (Rahbek et al. 2006). Slope and aspect, in particular, reflect the magnitude and direction of terrain curvature change among grid cells (Amatulli et al. 2018). In turn, roughness compares the difference between a cell and its eight surrounding cells as a measure of landscape heterogeneity, with values near one implying a flat surface (Wilson et al. 2007). Although aspect is often interpreted as a fine‐scale descriptor of slope exposure, at the 0.5° resolution, it may reflect broad‐scale terrain orientation and relief structure rather than local microclimatic conditions. At continental scales, aspect‐derived gradients are commonly interpreted as proxies for large‐scale patterns of solar energy and terrain organization, rather than hillslope‐level exposure (Wilson and Gallant 2000).

### 2.6 Modeling Historical Climate (Climate Stability)

Climatically stable areas during glaciation events likely served as habitat refugia where animal and plant species have persisted through time (Prance 1982; Carnaval et al. 2009, 2014; Graham et al. 2010), favoring lineage and species diversification (Graham et al. 2006; Araújo et al. 2008). To estimate biome‐level historical climatic stability in each grid cell, we used a Random Forest regression algorithm (Breiman 2001) to perform a joint model of the distribution of Amazonia, Cerrado, and the Atlantic Rainforest at 31 time periods over the last 120 kyr. We used the centroid of each 0.5 × 0.5° cell as a presence point for each biome, which resulted in 2128 points for Amazonia, 843 points for the Cerrado, and 502 points for the Atlantic Rainforest.

The Random Forest regression performs a set of successive classifiers, each independent of the previous one, which minimizes overfitting (Breiman 2001). The algorithm also computes the importance of each variable for the final model via the mean decrease in model accuracy. Variables more important for the regression model had higher values of the mean decrease in accuracy (Cutler et al. 2007; Table S1). To train our model, we created 3288 pseudo‐absence points from areas in South America that do not comprise any of the three biomes included in this study. We implemented 500 classification trees per biome (Amazonia, Cerrado, and Atlantic Rainforest) for model regression and the square root of the number of variables (a default parameter in classification models; Breiman 2001) at each split. To define regions of climatic stability at the biome level, we projected the best‐fitting present‐day model into 31 time periods (with a time interval of 4 kyr) over the past 120 kyr, using the Hadley Centre Climate reconstructions (Singarayer and Valdes 2010; Fuchs et al. 2013). We then overlaid these models of former habitat distribution to create a continuous layer of climatic stability, where the stability value of each cell varied from 0 (maximum climatic instability) to 31 (maximum climatic stability). Our stability metric quantifies the consistency of biome identity predicted by the model across climate scenarios, rather than the absence of climatic change itself. Additional details of the Random Forest method, as well as results for long‐term climatic stability, are available in the biome classification modeling (Supporting Information: Material S1, Table S1 and Figures S1–S4). Furthermore, unlike approaches on the basis of climate variance or species‐level niche tracking, our stability metric reflects the temporal persistence of biome‐scale climatic suitability inferred from repeated paleo‐projections of present‐day domain distributions.

We used the following R packages to implement the analyses described above: spThin (Aiello‐Lammens et al. 2015) for spatial thinning; sp (Pebesma et al. 2026), rgdal (Bivand et al. 2023), rgeos (Bivand and Rundel 2023), and raster (Hijmans, van Etten, et al. 2025) to handle spatial data and derive slope, aspect, and roughness from elevation; and randomForest (Liaw and Wiener 2002) and dismo (Hijmans et al. 2025) for biome distribution modeling and projection. Trait‐based ordinations were performed with vegan (Oksanen et al. 2026) for redundancy analysis (RDA), and functional diversity metrics (Gower dissimilarities and functional dispersion) were computed with FD (Laliberté et al. 2023).

### 2.7 Statistical Analyses

We tested multicollinearity among variables using Variance Inflation Factors (VIF) and removed those variables with VIF higher than 3 (Zuur et al. 2010) in the models described below. We scaled the remaining variables to standardized units (mean = 0, variance = 1). The final models included five variables: four of them correspond to present‐day environmental and climatic variables (potential evapotranspiration, precipitation of the driest quarter, relief slope, and relief aspect) and one was a historical climate variable estimated with the Random Forest modeling (climatic stability).

We used a Conditional Autoregressive (CAR) model to test the effects of these five variables on taxonomic, phylogenetic, and functional diversity, while accounting for spatial autocorrelation, in each target biome. CAR models were selected because they explicitly model spatial dependence among residuals while allowing direct inference on environmental covariates, which is appropriate when spatial autocorrelation arises from unmeasured spatially structured processes rather than from the predictors themselves. (Lichstein et al. 2002; Haining 2003). To account for this spatial autocorrelation, CAR uses neighborhood matrices that specify the relationship between the response value at each location (i) and neighboring locations (j) by fitting a variance–covariance matrix among non‐independent spatial observations. Spatial neighborhoods were defined on the basis of adjacency among grid cells, such that each cell was considered a neighbor of immediately adjacent cells. CAR models were implemented assuming a Gaussian error structure, with spatial dependence modeled through a conditional autoregressive structure on the residuals. The residuals were then used to estimate the effect of environmental variables on biodiversity metrics (Dormann et al. 2007). We calculated *R* ^2^ on the basis of Kissling and Carl (2008), and the CAR framework was used to explicitly absorb residual spatial autocorrelation, ensuring that estimated relationships between environmental variables and diversity metrics were not driven by spatial non‐independence. Ordinary Least Squares (OLS) models were used only to estimate standardized explanatory values following Rangel et al. (2006), facilitating comparison among predictors.

We used the R packages ade4 (Dray et al. 2026), adespatial (Dray et al. 2023), foreign (R Core Team 2025), rgeos (Bivand and Rundel 2023), sp. (Pebesma et al. 2026), rgdal (Bivand et al. 2023), and raster (Hijmans, Phillips, et al. 2025) to run CAR and OLS models.

We also compared FD between ecotone and core areas within each biome. We defined ecotone cells as grid cells belonging to a focal biome that had at least one neighboring cell (8‐neighborhood, including diagonals) belonging to an adjacent biome. This was implemented by shifting each focal cell by one grid step in all directions and identifying those shifts that matched the set of cells in the neighboring biome. We defined core cells as focal‐biome cells located away from biome boundaries. Specifically, we excluded any focal cells within a buffer (three grid steps) of any cell belonging to a different biome, yielding a set of interior “core” cells less likely to be influenced by boundary conditions. We also considered different buffers (four or five steps), but the results were virtually the same. For each biome comparison (Atlantic Forest–Cerrado, Cerrado–Amazon, and Amazon–Cerrado), we quantified differences in FD using a permutation test. We computed the average FD value across ecotone cells and compared it to a null distribution generated by repeatedly sampling the same number of cells from the core set (9999 permutations; sampling with replacement only when the core set was smaller than the ecotone set).

## 3 Results

### 3.1 Spatial Patterns of Biodiversity

**FIGURE 1:**
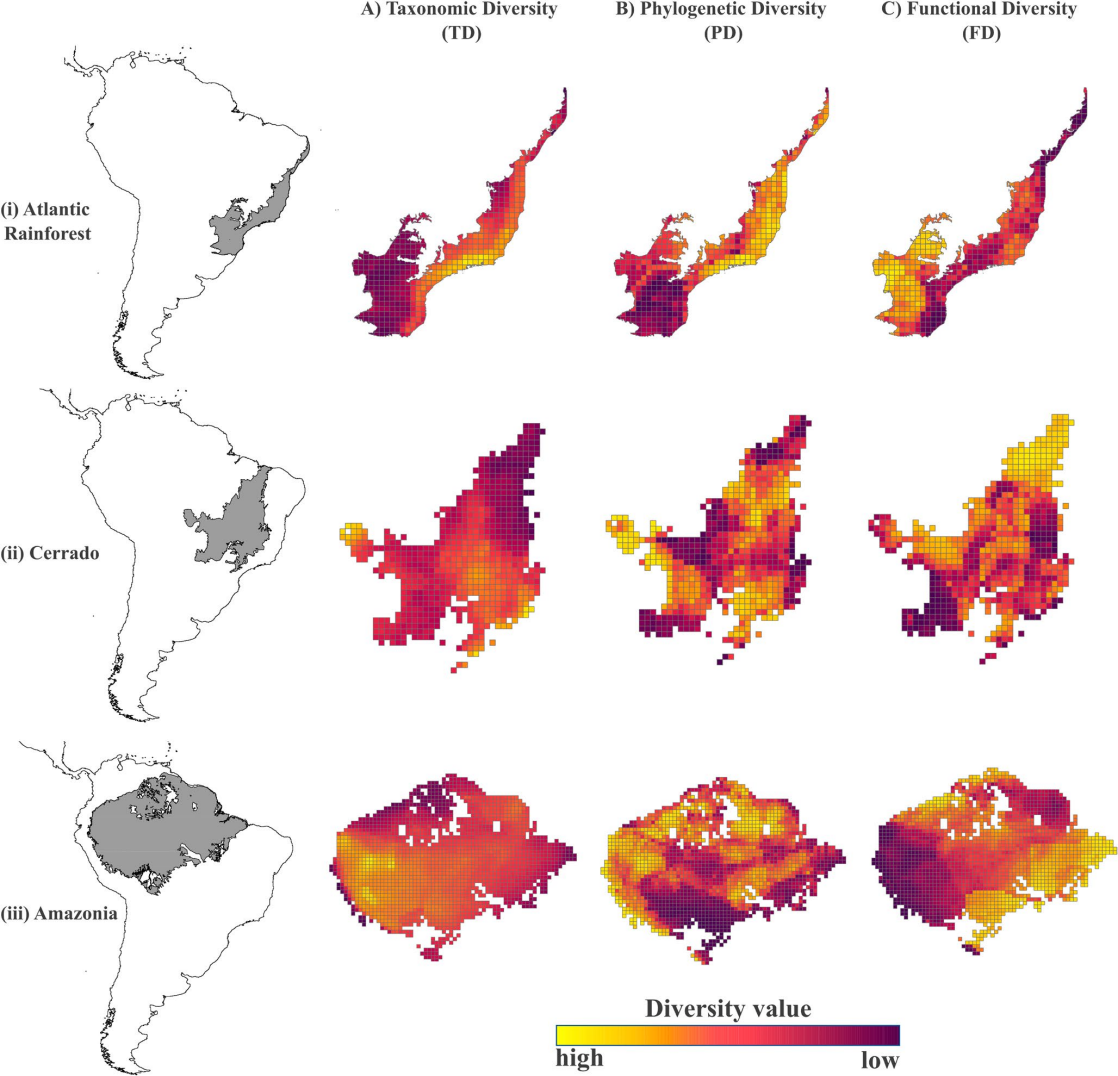
Spatial distribution of (A) Taxonomic Diversity (TD), (B) Phylogenetic Diversity (PD, quantified using the Net Relatedness Index, NRI), and (C) Functional Diversity (FD) across grid cells in the Atlantic Rainforest (i), Cerrado (ii), and Amazonia (iii). Color gradients represent relative diversity values, ranging from yellow (low diversity) to dark purple (high diversity).

We found *s*patial patterns of TD, PD, and FD to be heterogeneous within each biome (Figure 1). In the Atlantic Rainforest, TD is concentrated in the Serra do Mar mountains, along the eastern coast (Figure 1A‐i). There is a gradient of PD extending from the eastern into the northern portion of the Atlantic Rainforest biome (Figure 1B‐i). Conversely, high FD is found in the southwestern portion of the biome, which contacts the neighboring Cerrado and the southernmost biome of Brazil, called Pampa (Figure 1C‐ii and Figure S7). This finding highlights that ecotones, regions where adjacent biomes meet, concentrate trait diversity relative to the biome's core, a pattern that we have found for the Cerrado and the Atlantic Rainforest (see below; Figures S5 and S6). However, the Amazon core is an exception as it holds more FD than the ecotone with Cerrado (Figure S6).

Within the Cerrado, we found higher TD in the southeast and west (Figure 1A‐ii). In turn, PD is more geographically spread out, with some concentration in the central‐northern, southwestern, and southeastern Cerrado (Figure 1B‐ii). Higher FD is concentrated in the north, along a transitional area between the Cerrado, Amazonia, and Caatinga (Figure 1C‐ii).

In Amazonia, higher TD and PD were inferred in the northern and western regions (Figure 1A,B‐iii). However, PD is also high in Amazonia's central‐eastern region (Figure 1B‐iii). As in the Atlantic Rainforest and Cerrado, peripheral zones along Amazonia's edges present the highest values of FD relative to the biome's core (Figure 1C‐iii).

### 3.2 Predictors of Biodiversity Metrics

**FIGURE 2:**
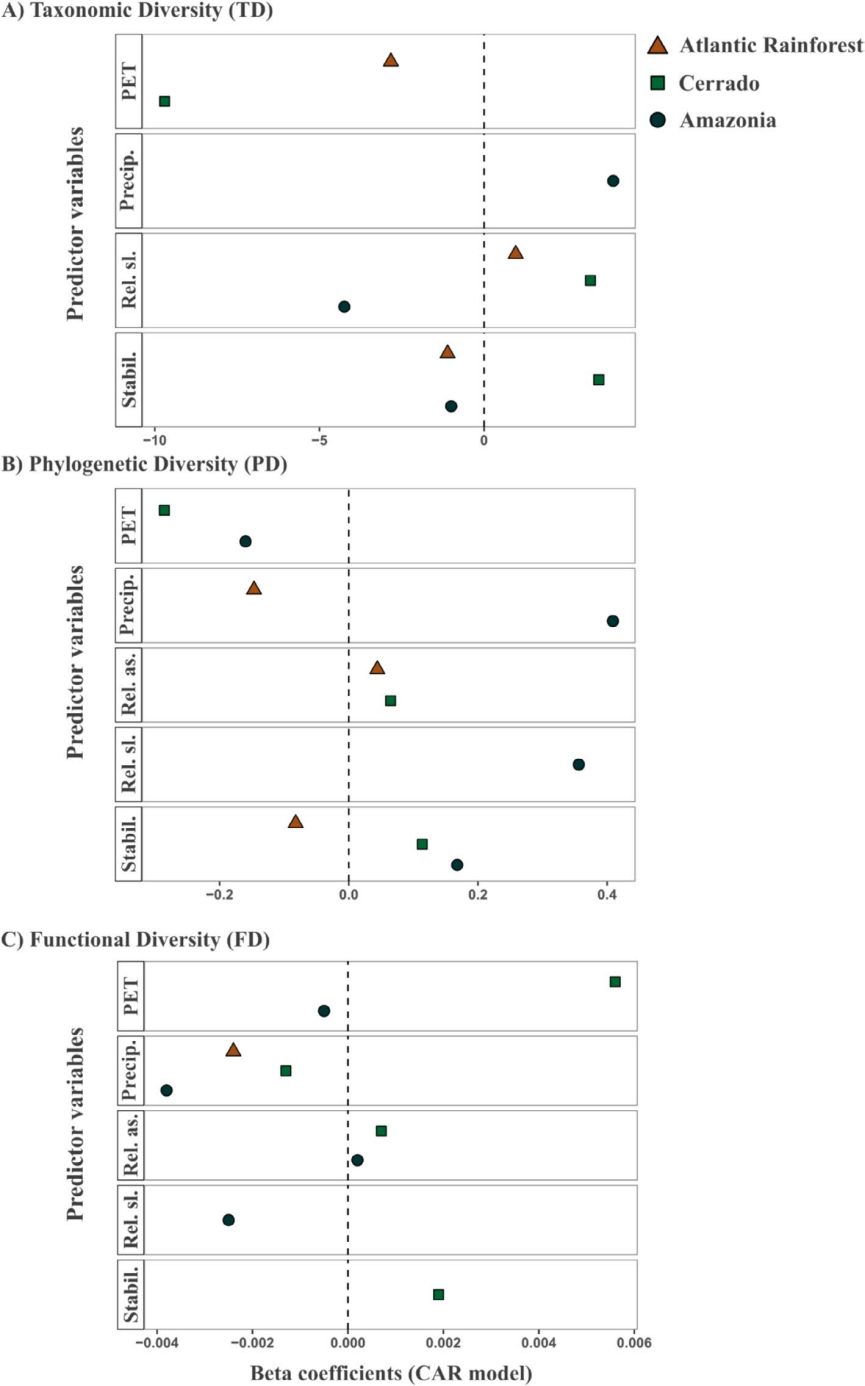
Beta coefficients from conditional autoregressive (CAR) models representing conditional associations between climatic and topographic predictors, climatic stability (Stabil.), relief slope (Rel. sl.), relief aspect (Rel. as.), precipitation of the driest quarter (Precip.), and potential evapotranspiration (PET), and biodiversity metrics: (A) Taxonomic Diversity (TD), (B) Phylogenetic Diversity (PD), and (C) Functional Diversity (FD). Positive and negative coefficients indicate the direction of associations, whereas the coefficient magnitude reflects the relative strength of predictors within each biome and diversity dimension. All predictors were standardized prior to model fitting; therefore, coefficients represent comparable effect sizes. Spatial structure is accounted for by the CAR framework and is not explicitly mapped in this figure.

Certain ecological and historical variables had similar effects on anuran diversity in all three biomes considered (Tables 1 and S5). Namely, both TD and PD increased with long‐term climatic stability in all regions. By contrast, precipitation of the driest quarter had an overall negative effect on FD (Tables 1 and S5, Figure 2).

**TABLE 1:** Standardized beta coefficients and associated significance levels from conditional autoregressive (CAR) models assessing the effects of environmental predictors on three biodiversity metrics—taxonomic diversity (TD), phylogenetic diversity (PD, quantified using the Net Relatedness Index, NRI), and functional diversity (FD)—across the Amazon, Cerrado, and Atlantic Rainforest. Statistically significant effects (*p* < 0.05) are shown in bold. PET denotes potential evapotranspiration.

Biome	Biodiversity metric	Variables	Beta	*p*
Atlantic Rainforest	TD	Precipitation	1.7922	0.0953
		PET	−9.7029	**< 0.001**
		Relief aspect	0.8558	0.153
		Relief slope	3.2301	**< 0.001**
		Stability	3.4856	**< 0.001**
	PD	Precipitation	−0.0707	0.149
		PET	−0.2856	**< 0.001**
		Relief aspect	0.0648	**0.018**
		Relief slope	0.0045	0.890
		Stability	0.1138	**< 0.001**
	FD	Precipitation	−0.0013	**0.016**
		PET	0.0056	**< 0.001**
		Relief aspect	0.0007	**0.026**
		Relief slope	−0.0004	0.244
		Stability	0.0019	**< 0.001**
Cerrado	TD	Precipitation	0.2157	0.544
		PET	−2.8302	**< 0.001**
		Relief aspect	−0.0437	0.777
		Relief slope	0.9608	**< 0.001**
		Stability	−1.1078	**0.001**
	PD	Precipitation	−0.1467	**< 0.001**
		PET	−0.0505	0.086
		Relief aspect	0.0442	**< 0.001**
		Relief slope	−0.0087	0.644
		Stability	−0.0822	**0.004**
	FD	Precipitation	−0.0024	**< 0.001**
		PET	0.0003	0.414
		Relief aspect	−0.0001	0.623
		Relief slope	−0.0003	0.118
		Stability	0.0000	0.974
Amazon	TD	Precipitation	3.9227	**< 0.001**
		PET	0.4953	0.160
		Relief aspect	−0.0041	0.980
		Relief slope	−4.2447	**< 0.001**
		Stability	−0.9978	**0.017**
	PD	Precipitation	0.4090	**< 0.001**
		PET	−0.1598	**< 0.001**
		Relief aspect	−0.0219	0.112
		Relief slope	0.3562	**< 0.001**
		Stability	0.1679	**< 0.001**
	FD	Precipitation	−0.0038	**< 0.001**
		PET	−0.0005	**0.019**
		Relief aspect	0.0002	**0.017**
		Relief slope	−0.0025	**< 0.001**
		Stability	−0.0002	0.403

**FIGURE 3:**
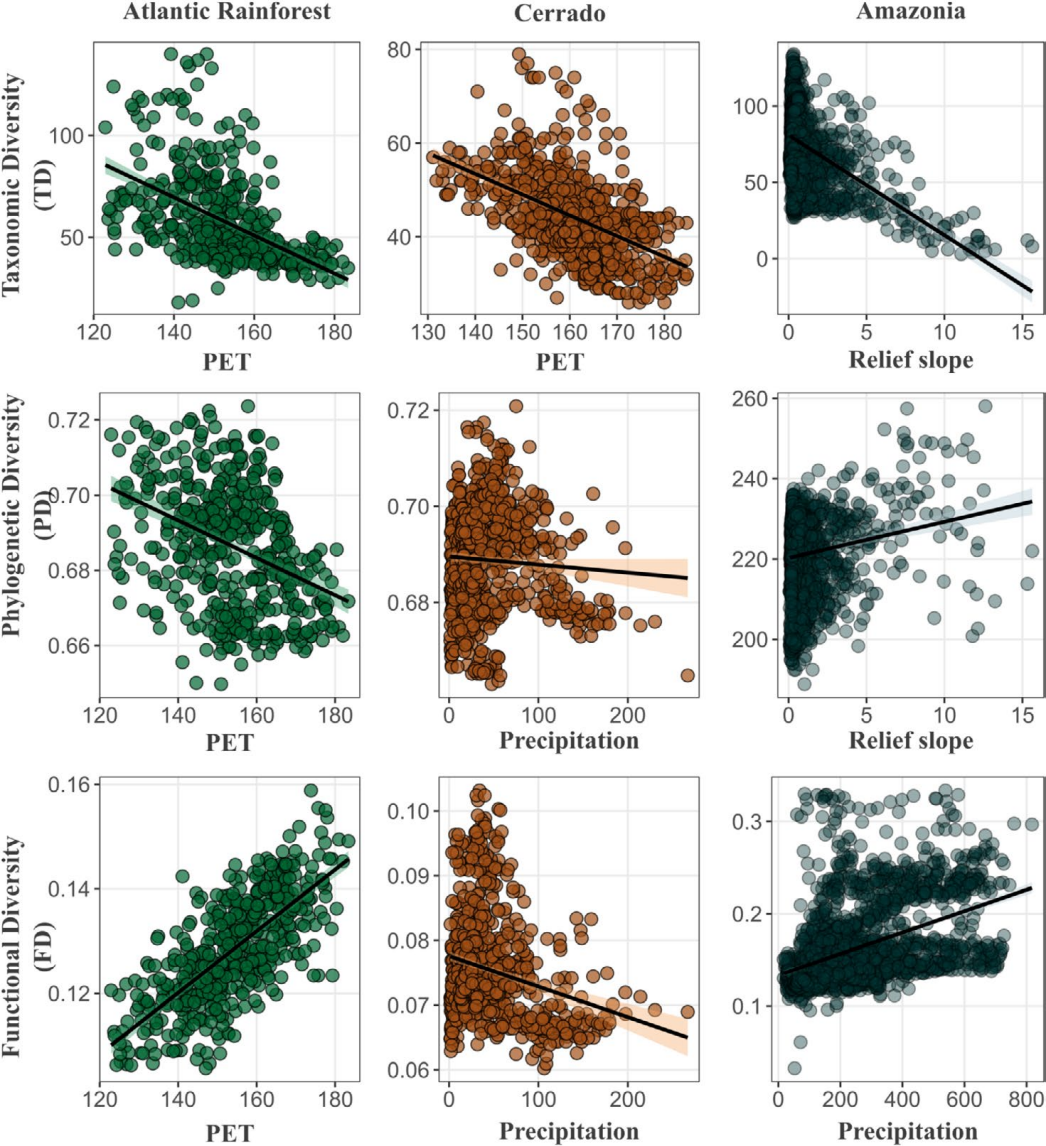
Relationships between key predictor variables, potential evapotranspiration (PET), precipitation of the driest quarter (bio17), relief slope, and Taxonomic Diversity (TD), Phylogenetic Diversity (PD), and Functional Diversity (FD).

Other variables had region‐specific effects on biodiversity. In the Atlantic Rainforest, PET is positively associated with FD, but negatively with TD and PD (Tables 1 and S5, Figures 2, 3). In Amazonia, precipitation has a positive correlation with FD, and topographic relief slope has a negative and positive correlation with PD and TD, respectively (Tables 1 and S5, Figures 2, 3). In contrast, in the Cerrado, precipitation has a negative correlation with PD and FD, and PET has a negative correlation with TD (Tables 1 and S6, Figures 2, 3).

Other variables have largely idiosyncratic effects across biodiversity metrics. For example, climatic stability has a negative association with TD and PD, but not with FD in the Cerrado (Table 1).

## 4 Discussion

### 4.1 General Spatial Biodiversity Patterns

Our analyses show that biological responses to contemporary and historical environmental conditions can be biome‐specific, and, sometimes, specific to a given biodiversity metric within a biome. We detected higher congruence in the environmental variables associated with biodiversity among the rainforest biomes, Amazonia, and the Atlantic Rainforest. In both regions, PET, topographic heterogeneity and current climate (precipitation of the driest quarter) were the main drivers of TD, PD, and TD. Shared landscape features in both biomes, such as the presence of a long mountain chain spanning the forest range—the Atlantic Rainforest *serras* and the Andes in the Amazonian region—might account for these shared responses (e.g., Schluter and Pennell 2017; Antonelli et al. 2018). Both mountain chains have been widely linked to species diversification, contributing lineages to adjacent lowland regions (Moraes et al. 2022; Prates et al. 2025; Vacher et al. 2024).

In contrast, and contrary to our predictions, PD and TD in the Cerrado were negatively correlated with climate stability. Two factors might explain this finding. First, our analyses might be missing a more important aspect of Cerrado ecology: the role of natural fire dynamics on regional biodiversity. Some studies suggest that fire dynamics generates a mosaic in the distribution of Cerrado species, as demonstrated for anurans (Valdujo et al. 2012), birds (Silva 1997), mammals (Costa 2003), woody plants (Klink and Machado 2005), and squamates (Nogueira et al. 2009). Second, being a mosaic of vegetation types, the Cerrado might be under a stronger influence of the species pools from neighboring biomes (Valdujo et al. 2012). Species colonizations from adjacent biomes into ecologically similar components of the Cerrado mosaic might swamp the effect of the environmental correlates tested here (see, e.g., Vasconcelos et al. 2014; Carmignotto et al. 2022).

### 4.2 Ecotones and Functional Diversity

A potential role for transitional ecotonal regions on biodiversity patterns seems particularly strong when considering FD. Our analyses reveal that most amphibian trait diversity accumulates in ecotones, except for the Amazon biome. In the absence of increased PD in ecotones relative to biome cores, ecotones seem to allow for the accumulation of unique traits but not necessarily lineages. Increased FD at biome edges suggests that environmental mosaics favor the accumulation of adaptive (trait) diversity within relatively narrower spatial bands. In particular, transitional zones might favor the co‐occurrence of complementary species sets owing to traits adapted to different environmental regimes, favoring niche complementarity (Hooper et al. 2005; Liautaud et al. 2020). Increased richness and the presence of unique species assemblages in ecotones have been previously characterized as typical of ecotones (Odum 1953). Our study highlights that this effect extends to FD, particularly between seasonally dry (Cerrado) and forested (Amazonian and Atlantic Rainforests) biomes.

### 4.3 Amazonia

Our findings show that variables describing both historical conditions (climate stability), habitat heterogeneity (relief slope), and present‐day environments (precipitation of the driest quarter and PET) have complementary roles in explaining amphibian species assemblages in Amazonia. This result is consistent with studies of birds (Chaves et al. 2011) and plants (Richardson et al. 2001; Antonelli et al. 2009), but contrasts with findings for treefrogs of the Hylidae family, whose local species richness has been linked to the timing of colonization of each Amazonian region (Wiens et al. 2011). The diversification of Amazonian anuran lineages has been associated with the topographic complexity created by the Andean uplift, with most peaks forming from the late middle Miocene to the early Pliocene (~7 to ~2 mya) (Gregory‐Wodzicki 2000; Mora et al. 2009; Vacher et al. 2024). This complexity, along with climate‐mediated landscape changes and the transformation of drainages associated with the Andean orogeny, has served as a significant driver of vicariance, contributing to the unique species richness in this region (Cracraft et al. 2020; Réjaud et al. 2020; Vacher et al. 2024). We further argue that the diversity of lineages was maintained over time because of climate stability, which was supported by consistently high levels of precipitation through time (Prance 1982; Hooghiemstra and van der Hammen 1998; Arruda et al. 2018). This stable precipitation likely drove the prevalence of species with reproductive modes independent of water bodies.

In contrast to PD, FD is higher in areas where precipitation and relief heterogeneity decrease in Amazonia. Lower precipitation and smoother topography characterize peripheral areas in Amazonia, where rainforest faunas come into contact with savanna ones. The encounter of species adapted to distinct environmental regimes may explain higher functional diversity in these areas.

### 4.4 Atlantic Rainforest

In the Atlantic Rainforest, we found that PET and climatic stability were the main correlates of the accumulation of multiple diversity metrics. PET had a negative impact on TD and PD. The explanation may reside in the hump‐shaped curve describing the well‐known pattern of species richness decreasing past an optimal value of PET, a measure usually associated with productivity (Tilman 1982; Abramsky and Rosenzweig 1984). FD, on the contrary, increased in sites with higher potential evapotranspiration and in ecotonal regions adjacent to the Cerrado biome.

Regarding the low but recurrent influence of climatic stability on biodiversity metrics in the Atlantic Rainforest, recent studies have detected a positive relationship between stability and TD, FD, and PD (da Silva et al. 2014). Compared to other regions, the relative stability to which many diversity hotspots have been exposed over evolutionary timescales (Fordham et al. 2019) has allowed for the accumulation and persistence of high species diversity (e.g., Araújo et al. 2008). Our results agree with the hypothesis that climate‐mediated persistence in habitat distributions and environmental conditions favors species diversification, low extinction, and accumulation of lineages with divergent functional traits. For instance, high FD in climatically stable regions of the Atlantic Forest may reflect the high diversity of anuran reproductive modes documented for this region, which allows the exploitation of the varied aquatic and humid microhabitats present (Haddad and Prado 2005). Long‐term stability of high precipitation and humidity regimes may have favored the diversification and regional accumulation of diverse reproductive strategies while potentially limiting competition for reproductive resources among amphibian species (Rodríguez et al. 2005).

Contrary to our expectations and to Rossa‐Feres et al. (2017), we did not find an influence of topographic slope on any metric of diversity in the Atlantic Forest. Rossa‐Feres et al. (2017) found the slope explained much of the variation found in the distribution pattern of anuran species richness in the Atlantic Rainforest, especially when compared to temperature and rainfall. Nevertheless, we found that the direction of the topographic relief (aspect) was associated with increased lineage and trait diversity. Aspect is expected to influence net primary production, solar energy irradiance, and biomass accumulation locally, which might favor long‐term species and trait diversification in our analysis. We acknowledge that the use of aspect at coarse spatial resolution is a limitation of our study, especially in the Atlantic Forest, where fine‐scale topographic effects may be important.

### 4.5 Cerrado

We expected that topographic heterogeneity in the Cerrado would correlate more strongly with diversity patterns. Instead, climatic stability was the most frequent, though not the strongest, driver of diversity patterns across all biodiversity metrics in this biome. This result aligns with studies showing that the high species richness (e.g., Werneck 2011), genetic diversity (Vasconcellos et al. 2019), and endemism (Azevedo et al. 2016) of several taxonomic groups in Cerrado plateaus can be explained by climatic stability during the Quaternary period. In turn, we found precipitation and productivity to have a negative relationship with biodiversity metrics in the Cerrado. This pattern might reflect the presence of many anuran species with behaviors and reproductive modes adapted to drier climates (Haddad and Prado 2005; Ceron et al. 2020) or reproductive periods restricted to the rainy season (Pereira et al. 2020). Finally, heterogeneous responses of each biodiversity metric to environmental predictors might reflect a higher influence of species pools that expanded into the Cerrado from adjacent biomes (Valdujo et al. 2012) and the Cerrado's fire‐drive habitat heterogeneity (Silva et al. 2006; Nogueira et al. 2009). Future investigations should focus on quantifying these effects, which remains a challenging task.

## 5 Conclusion

Our analyses support a general pattern where different environmental variables contribute more strongly to variation in the geographic distribution of different metrics of biodiversity: TD, FD, and PD. We found a higher degree of congruence in variable importance between biomes with similar structural characteristics (e.g., rainforests), but also inferred biome‐specific effects. In turn, certain environmental variables had consistent effects on biodiversity, as is the case with historical climate stability, which increased all biodiversity metrics. Finally, we found that ecotones hold relatively higher FD, suggesting that transitional zones between biomes with distinct characteristics (e.g., between forests and savannas) favors the local accumulation of diverse ecologies and underlying organismal traits.

Although these findings underscore the unique and complementary roles that historical and present‐day environmental conditions play in shaping biodiversity, it is crucial to acknowledge the limitations inherent to our study. For example, the exclusion of variables describing fire dynamics (in the Cerrado) or microclimatic variations (in ecotones) might affect our results and interpretations. Future analyses would also benefit from accounting for uncertainty in the inference of phylogenetic trees used to estimate PD. Addressing these aspects in future studies could refine our understanding of the patterns and processes at play. Such a comprehensive understanding of how ecological and historical factors correlate to distinct metrics of biodiversity in forests and savannas around the world will continue to provide insights and generalizations about the mechanisms underlying community assembly and the heterogeneous distribution of life on Earth.

## Author Contributions

**Thiago Gonçalves‐Souza:** conceptualization (lead), data curation (supporting), formal analysis (lead), investigation (equal), methodology (equal). **Lilian S. O. Melo:** conceptualization (lead), data curation (lead), formal analysis (lead), methodology (lead). **Ana C. Carnaval:** conceptualization (equal), formal analysis (supporting), funding acquisition (equal), methodology (supporting). **Denise de C. Rossa‐Feres:** conceptualization (lead), funding acquisition (lead), methodology (equal), project administration (lead). **Andrea Paz:** formal analysis (supporting), methodology (supporting). **Ivan Prates:** data curation (equal), formal analysis (lead), investigation (equal), methodology (equal).

## Funding

This work was supported by Fundação de Amparo à Pesquisa do Estado de São Paulo.

## Conflicts of Interest

The authors declare no conflicts of interest.

## Supporting information


**Figure S1:** Present‐day random forest classification modeling for the Amazon, the Cerrado, and Atlantic Rainforest.
**Table S1:** Variable importance measures on the basis of mean decrease in accuracy and mean decrease in Gini index. Higher mean decreases in accuracy values indicate greater relative importance of the predictor.
**Figure S2:** Maps of the Atlantic Rainforest predictor variables distribution. Precipitation of the driest quarter (BIO17), Mountain Aspect (Aspect), Terrain slope (relief slope), Potential Evapotranspiration of the Warmest Quarter (PET warm), and Climatic stability over the past 120,000 years (Climatic Stability).
**Figure S3:** Maps of the Cerrado predictor variables distribution. Precipitation of the driest quarter (BIO17), Mountain Aspect (Aspect), Terrain slope (relief slope), Potential Evapotranspiration of the Warmest Quarter (PET warm), and Climatic stability over the past 120,000 years (Climatic Stability).
**Figure S4:** Maps of the Amazon predictor variables distribution. Precipitation of the driest quarter (BIO17), Mountain Aspect (Aspect), Terrain slope (relief slope), Potential Evapotranspiration of the Warmest Quarter (PET warm), and Climatic stability over the past 120,000 years (Climatic Stability).
**Table S2:** Imputed trait dataset according to Brownian evolutionary model for 1090 species present in the Amazon, the Atlantic Rainforest and the Cerrado. Snout‐vent length (SVL), head width and tibia length are represented in millimeter.
**Table S3:** Species traits selected and included in the functional analyses per 0.5 × 0.5 grid for the Amazon, the Cerrado, and the Atlantic Rainforest. We provided the biological interpretation of each trait as well as its ecological function for a clearer interpretation of the reason for including each one of them.
**Table S4:** Phylogenetic signal of continuous imputed traits across the three domains. Phylogenetic signal was quantified using Blomberg's K statistics (K), with statistical significance assessed via phylogenetically independent contrasts (PIC; *p*‐value and Z‐score). Values of *K* < 1 indicate a weaker phylogenetic signal than expected under Brownian motion, *K* = 1 indicates evolution consistent with Brownian motion, and *K* > 1 indicates a stronger phylogenetic signal.
**Table S5:** Phylogenetic signal for imputed categorical data in the three biomes. The traits show phylogenetic signal when the observed number of changes in trait states is lower than the median null number of changes. *p* is the significance value.
**Table S6:** Summary of Conditional Autoregressive (CAR) model results for the influence of the topographic heterogeneity (Relief aspect and slope), climatic stability, and current climate variables (precipitation of the driest quarter, bio17) in each biodiversity dimension. The beta coefficients and their significance are available in Table 1 in the main text.
**Figure S5:** Spatial patterns of Functional Diversity (as in Figure 1, main text) emphasizing higher functional diversity (arrows pointing to yellowish grid cells) in adjacent, transitional zones (ecotones).
**Figure S6:** Differences in functional diversity (FD) between ecotone grid cells (cells adjacent to a neighboring biome) and core grid cells (interior cells of the same biome), shown as mean effects (points) with 95% bootstrap confidence intervals (lines); the dashed vertical line indicates no difference (0). Positive values (blue) indicate higher functional diversity in ecotone grid cells than in the biome core, whereas negative values (green) indicate higher functional diversity in the biome core than in ecotone grid cells.
**Figure S7:** Spatial distribution of terrestrial Brazilian biomes. Biome boundaries were obtained from official IBGE datasets using the geobr R package (Pereira and Gonçalves 2024), doi:10.32614/CRAN.package.geobr.

## Data Availability

Data openly available in a public repository https://zenodo.org/records/15685449.
