# Supplementary material for: Ecological and Historical Correlates of Taxonomic, Phylogenetic, and Functional Diversity of Amphibians in South American Rainforests and Savannas
Source: Ecol Evol. 2026 Apr 16;16(4):e73494. doi: 10.1002/ece3.73494 (PMC13086635; doi:10.1002/ece3.73494)
Supplement: Supplementary file 1 — Figure S1: Present‐day random forest classification modeling for the Amazon, the Cerrado, and Atlantic Rainforest. Table S1: Variable importance measures on the basis of mean decrease in accuracy and mean decrease in Gini index. Higher mean decreases in accuracy values indicate greater relative importance of the predictor. Figure S2: Maps of the Atlantic Rainforest predictor variables distribution. Precipitation of the driest quarter (BIO17), Mountain Aspect (Aspect), Terrain slope (relief slope), Potential Evapotranspiration of the Warmest Quarter (PET warm), and Climatic stability over the past 120,000 years (Climatic Stability). Figure S3: Maps of the Cerrado predictor variables distribution. Precipitation of the driest quarter (BIO17), Mountain Aspect (Aspect), Terrain slope (relief slope), Potential Evapotranspiration of the Warmest Quarter (PET warm), and Climatic stability over the past 120,000 years (Climatic Stability). Figure S4: Maps of the Amazon predictor variables distribution. Precipitation of the driest quarter (BIO17), Mountain Aspect (Aspect), Terrain slope (relief slope), Potential Evapotranspiration of the Warmest Quarter (PET warm), and Climatic stability over the past 120,000 years (Climatic Stability). Table S2: Imputed trait dataset according to Brownian evolutionary model for 1090 species present in the Amazon, the Atlantic Rainforest and the Cerrado. Snout‐vent length (SVL), head width and tibia length are represented in millimeter. Table S3: Species traits selected and included in the functional analyses per 0.5 × 0.5 grid for the Amazon, the Cerrado, and the Atlantic Rainforest. We provided the biological interpretation of each trait as well as its ecological function for a clearer interpretation of the reason for including each one of them. Table S4: Phylogenetic signal of continuous imputed traits across the three domains. Phylogenetic signal was quantified using Blomberg's K statistics (K), with statistical significance assessed via ph [file ECE3-16-e73494-s001.docx]

**SUPPORTING INFORMATION**

**Ecological and historical drivers of taxonomic, phylogenetic, and functional diversity of amphibians in South American rainforests and savannas**

Thiago Gonçalves-Souza, Lilian S. O. Melo, Ana C. Carnaval, Denise de C. Rossa-Feres, Andrea Paz & Ivan Prates

**Content:**

**Supporting Information: Material S1.**

Figure S1

Table S1

**Supporting Information: Material S2.**

Figure S2

Figure S3

Figure S4

Table S2

**Supporting Information: Material S3.**

Table S2

Table S3

Table S4

Table S5

Figure S5

Figure S6

Figure S7

**Supporting Information: Material S1. Biome classification modelling**

The present-day domain classification modelling achieved through Random Forest was similar to present-day distribution maps of the Atlantic Rainforest, the Cerrado, and the Amazon (Figure S1). The mean prediction error of the present-day domain modeling was estimated as 22.02%. The model did not classify either the transitional area located between the Amazon and the Cerrado (*babaçu* forest), or the dry patches within the Amazon, as either Cerrado or Amazon. Yet, it classified the northern range of the coastal Atlantic Rainforest as belonging to the Amazon domain. Therefore, we have taken out those mispredictions from the Atlantic Rainforest stability maps. Precipitation of the driest quarter, mean temperature of the coldest quarter, and the mean temperature of the warmest quarter were the most important variables guiding the final habitat classification model (Table S1).


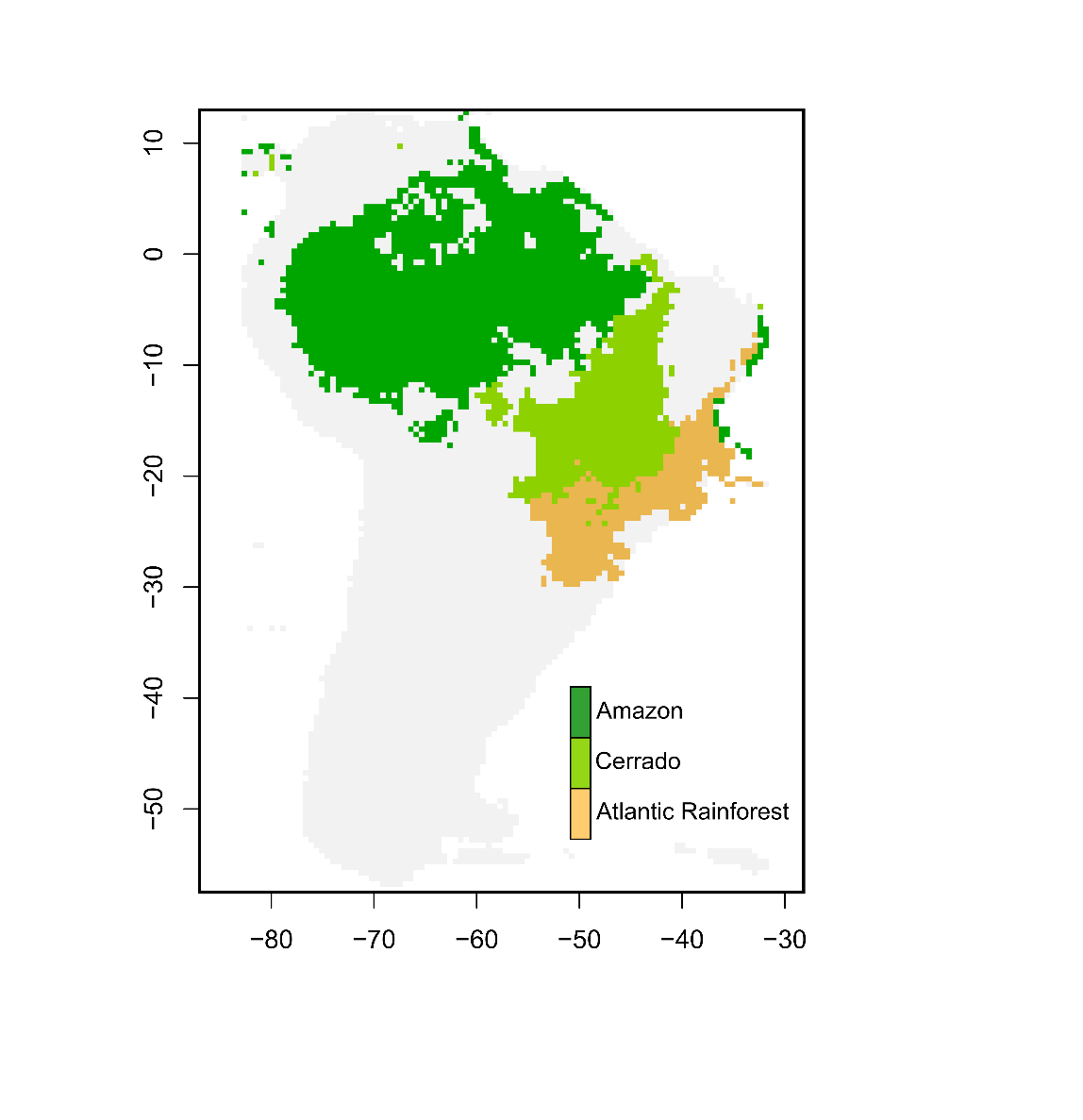


**Figure S1**. Present day random forest classification modelling for the Amazon, the Cerrado and Atlantic Rainforest.

**Table S1**. Variable importance measures based on mean decrease in accuracy and mean decrease in Gini index. Higher mean decreases in accuracy values indicate greater relative importance of the predictor.

| Climatic variable | Mean Decrease Accuracy | Mean Decrease Gini |
| --- | --- | --- |
| bio1 | 43.46 | 512.62 |
| **bio10** | **58.81** | 470.44 |
| **bio11** | **58.9** | 707.2 |
| bio12 | 51.43 | 899.6 |
| bio16 | 38.29 | 830.64 |
| **bio17** | **129.47** | 606 |

**Supporting Information: Material S2. Mapping habitat stability**

When projected into the past and summed up the stability of each 4 kyr period, the final domain stability map showed stable areas that are congruent with highlands (mountains and plateaus) in the Atlantic Rainforest and Cerrado. In contrast, the stable areas in the Amazon are associated with the lowland areas along the Amazon river basin.

In the Atlantic Rainforest and Cerrado we observed two widespread climatic stable areas with a narrow disjunction separating each of them. Both climatically stable areas showed low internal variation. In the Atlantic Rainforest, those separated stable areas are in the central-south portion of the domain (Figure S2). The occurrence of the climatic stable areas in the Cerrado is associated with the central-southwestern and the central-northeastern plateaus (Figure S3), which even with the disjunction, preserved a small connection between them in the center of Cerrado. Besides the most climatic stable areas be present widespread along both sides in the Amazon river without disjunction, they occurred from the base of the Andes following Marañón river course extending across the course of Amazon River until central eastside of Amazon with low internal variation (Figure S4). Two portions of climatic stability are observed extending from the Amazon river to the north, and the only two disjunction portions of climatic stable areas are located south and north of the Amazon river downstream in the Atlantic Ocean (Figure S4).


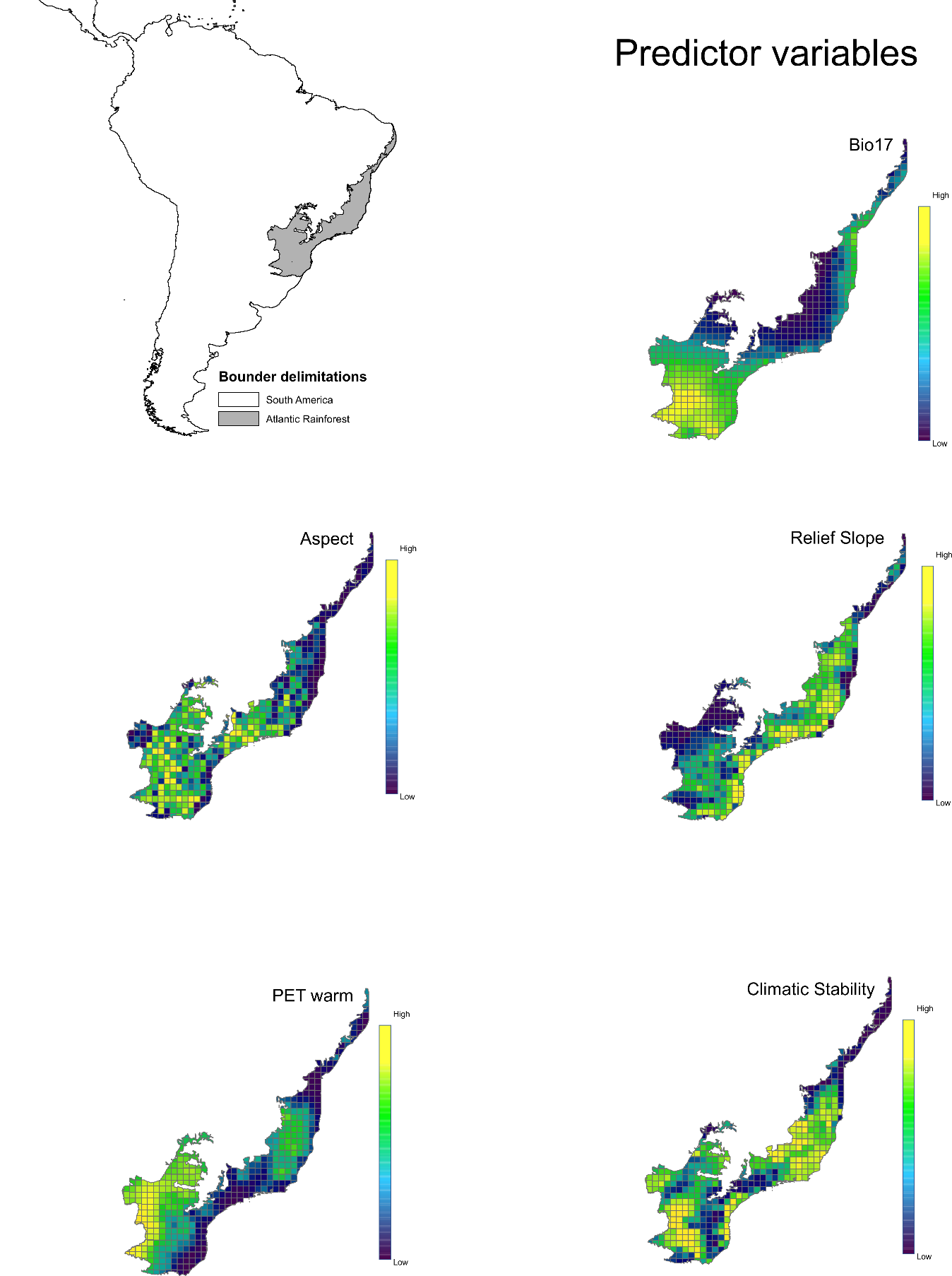


**Figure S2**. Maps of the Atlantic Rainforest predictor variables distribution. Precipitation of driest quarter (BIO17), Mountain Aspect (Aspect), Terrain slope (relief slope), Potential Evapotranspiration of the Warmest Quarter (PET warm), Climatic stability over the past 120,000 years (Climatic Stability).


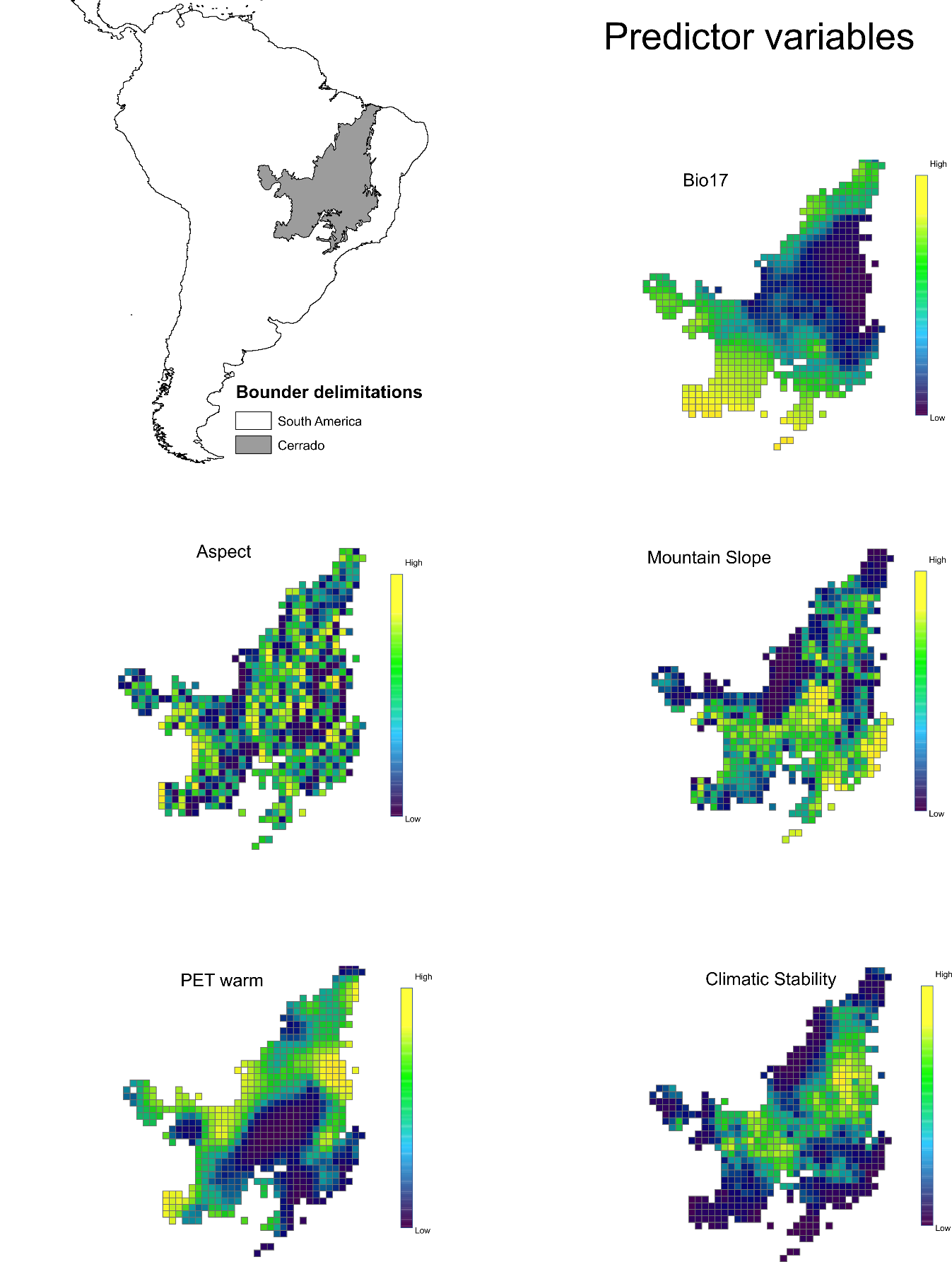


**Figure S3**. Maps of the Cerrado predictor variables distribution. Precipitation of driest quarter (BIO17), Mountain Aspect (Aspect), Terrain slope (relief slope), Potential Evapotranspiration of the Warmest Quarter (PET warm), Climatic stability over the past 120,000 years (Climatic Stability).


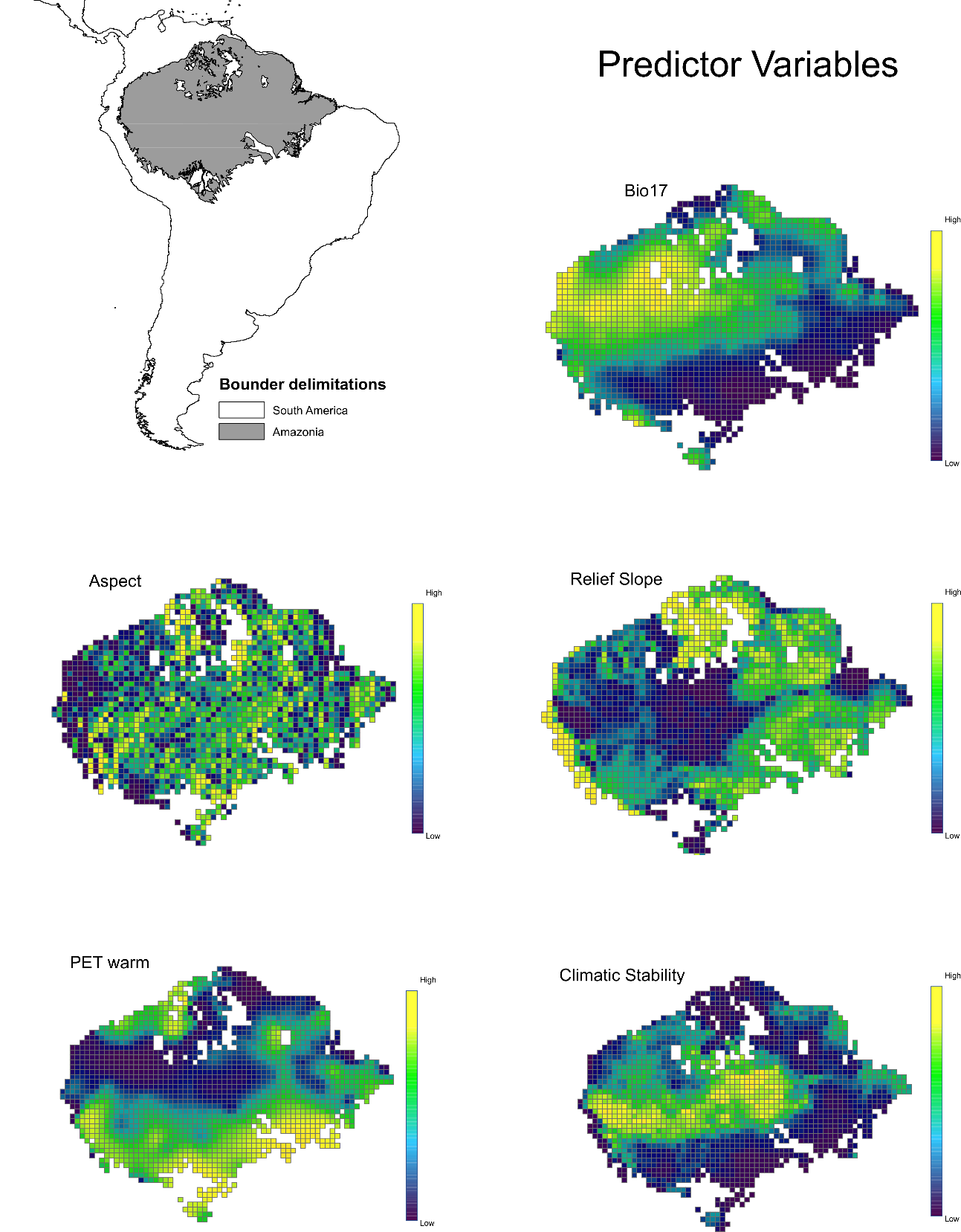


**Figure S4**. Maps of the Amazon predictor variables distribution. Precipitation of driest quarter (BIO17), Mountain Aspect (Aspect), Terrain slope (relief slope), Potential Evapotranspiration of the Warmest Quarter (PET warm), Climatic stability over the past 120,000 years (Climatic Stability).

**Table S2**. Imputed trait dataset according to Brownian evolutionary model for 1090 species present in the Amazon, the Atlantic Rainforest and the Cerrado. Snout-vent length (SVL), head width and tibia length are represented in millimeter.

| Species | SVL | Head width | Tibia lengh |
| --- | --- | --- | --- |
| *Adelastes hylonomos* | 28.9 | 8.8 | 10 |
| *Adelophryne adiastola* | 14.6 | 4.64 | 6.2 |
| *Adelophryne gutturosa* | 13 | 4.5 | 6 |
| *Adelophryne pachydactyla* | 11.1 | 4.32 | 5.46 |
| *Adelophryne patamona* | 21.5 | 8.5 | 11 |
| *Adelphobates castaneoticus* | 21.9 | 7.03 | 8.78 |
| *Adelphobates galactonotus* | 34.7 | 12.05 | 15.27 |
| *Adelphobates quinquevittatus* | 18.98 | 6.09 | 8.5 |
| *Adenomera ajurauna* | 19.03 | 7.09 | 8.15 |
| *Adenomera andreae* | 21 | 9 | 13.04 |
| *Adenomera araucaria* | 19.9 | 6.7 | 9.34 |
| *Adenomera bokermanni* | 24 | 7.9 | 10.4 |
| *Adenomera diptyx* | 22 | 7.61 | 10.27 |
| *Adenomera heyeri* | 25.8 | 10.3 | 10.8 |
| *Adenomera hylaedactyla* | 23.48 | 8.61 | 10.92 |
| *Adenomera lutzi* | 30.1 | 11.2 | 14.6 |
| *Adenomera marmorata* | 21.9 | 6.69 | 9.2 |
| *Adenomera martinezi* | 23 | 7 | 11.09 |
| *Adenomera nana* | 18.9 | 6.09 | 8.88 |
| *Adenomera thomei* | 23.21 | 6.75 | 10.17 |
| *Agalychnis buckleyi* | 52.4 | 17 | 26.8 |
| *Agalychnis hulli* | 37.1 | 14.1 | 18.6 |
| *Allobates alessandroi* | 22 | 7.6 | 10.2 |
| *Allobates brunneus* | 18 | 5.5 | 8.5 |
| *Allobates caeruleodactylus* | 16.1 | 5.2 | 7.5 |
| *Allobates conspicuus* | 16 | 5.4 | 8 |
| *Allobates crombiei* | 17.7 | 6.2 | 9.1 |
| *Allobates femoralis* | 25 | 8 | 12 |
| *Allobates fuscellus* | 17.5 | 5.7 | 8 |
| *Allobates gasconi* | 13.9 | 5 | 6.1 |
| *Allobates goianus* | 18 | 6 | 8.09 |
| *Allobates granti* | 16.8 | 5.29 | 7.63 |
| *Allobates insperatus* | 16.7 | 6.1 | 8.1 |
| *Allobates marchesianus* | 15.85 | 5.2 | 7.6 |
| *Allobates masniger* | 19 | 6.5 | 8.6 |
| *Allobates melanolaemus* | 22.7 | 7.3 | 10.6 |
| *Allobates myersi* | 32.8 | 10.5 | 15 |
| *Allobates nidicola* | 20.2 | 6.7 | 8.5 |
| *Allobates olfersioides* | 16.9 | 5.1 | 7.27 |
| *Allobates ornatus* | 17.3 | 5.3 | 8.7 |
| *Allobates paleovarzensis* | 20.06 | 5.85 | 9.21 |
| *Allobates subfolionidificans* | 17.6 | 6.4 | 8.3 |
| *Allobates sumtuosus* | 15.6 | 5.6 | 7.5 |
| *Allobates trilineatus* | 17.2 | 6 | 8 |
| *Allobates undulatus* | 22.2 | 6.8 | 10.1 |
| *Allobates vanzolinius* | 21.8 | 7.1 | 9.8 |
| *Allobates zaparo* | 28 | 9.35 | 13.28 |
| *Allophryne ruthveni* | 21 | 7 | 10.28 |
| *Amazophrynella bokermanni* | 22 | 7 | 10.5 |
| *Amazophrynella minuta* | 19.7 | 5.82 | 8.44 |
| *Ameerega bassleri* | 37.5 | 12.19 | 17.43 |
| *Ameerega berohoka* | 23 | 7 | 10.6 |
| *Ameerega bilinguis* | 30.78 | 9.52 | 14.32 |
| *Ameerega braccata* | 20.19 | 6.26 | 10.12 |
| *Ameerega cainarachi* | 27.85 | 8.2 | 14.65 |
| *Ameerega flavopicta* | 26.66 | 8.03 | 12.99 |
| *Ameerega hahneli* | 19.02 | 5.76 | 9.33 |
| *Ameerega ingeri* | 27.5 | 8.5 | 12 |
| *Ameerega macero* | 28.59 | 9 | 14.38 |
| *Ameerega parvula* | 28.5 | 8.75 | 12.87 |
| *Ameerega petersi* | 28 | 8.3 | 14.39 |
| *Ameerega picta* | 22.2 | 6.38 | 10.75 |
| *Ameerega planipaleae* | 26.5 | 7.89 | 12.1 |
| *Ameerega pongoensis* | 28.59 | 8.78 | 12.94 |
| *Ameerega pulchripecta* | 26 | 7.11 | 12.34 |
| *Ameerega rubriventris* | 21.1 | 6.6 | 9.9 |
| *Ameerega silverstonei* | 38.3 | 10.1 | 18.3 |
| *Ameerega simulans* | 27 | 7.7 | 12 |
| *Ameerega smaragdina* | 26.5 | 7.69 | 13.77 |
| *Ameerega trivittata* | 43.5 | 12 | 20.5 |
| *Anomaloglossus ayarzaguenai* | 26.6 | 9.2 | 13.6 |
| *Anomaloglossus baeobatrachus* | 17.6 | 5.5 | 7.9 |
| *Anomaloglossus beebei* | 17.8 | 6.1 | 8 |
| *Anomaloglossus degranvillei* | 20.1 | 7.8 | 9.9 |
| *Anomaloglossus guanayensis* | 21.5 | 8 | 11.5 |
| *Anomaloglossus kaiei* | 18.9 | 6.6 | 8.8 |
| *Anomaloglossus parimae* | 22 | 7.6 | 10.7 |
| *Anomaloglossus parkerae* | 17 | 6.4 | 9.9 |
| *Anomaloglossus shrevei* | 19.3 | 7.3 | 9.6 |
| *Anomaloglossus stepheni* | 16.63 | 5.97 | 7.98 |
| *Anomaloglossus tamacuarensis* | 22.4 | 8 | 11.7 |
| *Anomaloglossus tepuyensis* | 28.3 | 9.7 | 14 |
| *Anomaloglossus triunfo* | 19.7 | 6.55 | 9.65 |
| *Anomaloglossus wothuja* | 21.7 | 6 | 10.3 |
| *Aparasphenodon bokermanni* | 71.1 | 21.2 | 34 |
| *Aparasphenodon brunoi* | 49.16 | 20.5 | 25.64 |
| *Aparasphenodon venezolanus* | 49.82 | 14.24 | 22.2 |
| *Aplastodiscus albofrenatus* | 39.3 | 12.97 | 19.26 |
| *Aplastodiscus albosignatus* | 41.5 | 13.28 | 21.58 |
| *Aplastodiscus arildae* | 38.7 | 12.6 | 18.7 |
| *Aplastodiscus callipygius* | 48 | 16 | 23.2 |
| *Aplastodiscus cavicola* | 33.2 | 11.6 | 17.3 |
| *Aplastodiscus cochranae* | 43.56 | 16.65 | 20.69 |
| *Aplastodiscus ehrhardti* | 33.7 | 10.38 | 17.27 |
| *Aplastodiscus eugenioi* | 37.5 | 13 | 19.8 |
| *Aplastodiscus flumineus* | 45.1 | 14.3 | 23.6 |
| *Aplastodiscus ibirapitanga* | 41.3 | 13.3 | 19.3 |
| *Aplastodiscus leucopygius* | 42 | 13.8 | 20.5 |
| *Aplastodiscus musicus* | 47 | 16 | 22.64 |
| *Aplastodiscus perviridis* | 39.66 | 14.97 | 18.88 |
| *Aplastodiscus sibilatus* | 31.8 | 10.4 | 15.9 |
| *Aplastodiscus weygoldti* | 41.7 | 13.7 | 20.8 |
| *Arcovomer passarellii* | 16 | 3.44 | 4.04 |
| *Atelopus andinus* | 28 | 8 | 12.3 |
| *Atelopus boulengeri* | 51 | 15.5 | 22.44 |
| *Atelopus dimorphus* | 31.3 | 7.1 | 13.9 |
| *Atelopus flavescens* | 28.92 | 8.31 | 13.03 |
| *Atelopus franciscus* | 30.69 | 8.56 | 13.71 |
| *Atelopus pulcher* | 34.07 | 9.1 | 15.47 |
| *Atelopus seminiferus* | 40 | 9 | 16.73 |
| *Atelopus siranus* | 23.5 | 6.3 | 10.4 |
| *Atelopus spumarius* | 34.6 | 9.6 | 15.4 |
| *Atelopus tricolor* | 20.82 | 6.18 | 9.7 |
| *Barycholos ternetzi* | 30.4 | 10.2 | 17.2 |
| *Boana albomarginata* | 56 | 21 | 30 |
| *Boana albopunctata* | 58 | 17.4 | 31.9 |
| *Boana atlanticus* | 38.32 | 13.83 | 18.3 |
| *Boana balzani* | 48.5 | 16.6 | 26 |
| *Boana beckeri* | 32.8 | 10.5 | 16.3 |
| *Boana bischoffi* | 42.8 | 19.24 | 30.9 |
| *Boana boans* | 105 | 40 | 58.5 |
| *Boana buriti* | 30 | 7.7 | 15.1 |
| *Boana caingua* | 33.1 | 8.32 | 17.45 |
| *Boana calcarata* | 48.2 | 17.6 | 28.8 |
| *Boana cinerascens* | 35.3 | 12.24 | 18.21 |
| *Boana cipoensis* | 34.35 | 11.88 | 18.44 |
| *Boana crepitans* | 65 | 23 | 36 |
| *Boana cymbalum* | 47 | 14.75 | 25.1 |
| *Boana dentei* | 50 | 18 | 25.49 |
| *Boana ericae* | 31.8 | 10.8 | 16.9 |
| *Boana exastis* | 86.5 | 30.2 | 50.1 |
| *Boana faber* | 99.2 | 40.48 | 58.13 |
| *Boana fasciata* | 35.4 | 11.39 | 20.07 |
| *Boana freicanecae* | 40 | 15.3 | 21.7 |
| *Boana geographica* | 49 | 18 | 24 |
| *Boana goiana* | 33 | 10.93 | 18.71 |
| *Boana guentheri* | 36 | 10.4 | 16.74 |
| *Boana hobbsi* | 42.5 | 14.7 | 21.3 |
| *Boana hutchinsi* | 43 | 15.8 | 21.8 |
| *Boana jimenezi* | 30.9 | 11.43 | 16.69 |
| *Boana joaquini* | 50.86 | 16.69 | 27.33 |
| *Boana lanciformis* | 63.2 | 19.8 | 44.2 |
| *Boana latistriata* | 44.8 | 13.7 | 22 |
| *Boana lemai* | 31.5 | 12.3 | 16.7 |
| *Boana leptolineata* | 30 | 8.6 | 15.2 |
| *Boana leucocheila* | 75.4 | 22.6 | 46 |
| *Boana liliae* | 35.15 | 12.85 | 19.23 |
| *Boana lundii* | 72.4 | 24.5 | 37.5 |
| *Boana marginata* | 46.3 | 15.6 | 24.3 |
| *Boana microderma* | 31.7 | 11.1 | 16.2 |
| *Boana multifasciata* | 50.2 | 15.7 | 29.2 |
| *Boana nympha* | 32.7 | 11.5 | 17.3 |
| *Boana ornatissima* | 40.8 | 13.79 | 23.34 |
| *Boana palaestes* | 49 | 14.8 | 23.7 |
| *Boana pardalis* | 68.7 | 23.36 | 35.04 |
| *Boana phaeopleura* | 32.7 | 10 | 17.4 |
| *Boana polytaenia* | 38.1 | 11.81 | 19.05 |
| *Boana pombali* | 58.1 | 19.2 | 30.2 |
| *Boana prasina* | 44.7 | 13.86 | 22.8 |
| *Boana pulchella* | 43.5 | 14.01 | 22.07 |
| *Boana punctata* | 35.7 | 12.5 | 18.9 |
| *Boana raniceps* | 69 | 22 | 40 |
| *Boana rhythmica* | 32.9 | 11.3 | 17.3 |
| *Boana roraima* | 45.5 | 15.7 | 25.2 |
| *Boana secedens* | 56.4 | 17.46 | 26.8 |
| *Boana semiguttatus* | 43.1 | 14.3 | 22.7 |
| *Boana semilineata* | 68.3 | 24.43 | 34.8 |
| *Boana sibleszi* | 32.9 | 12.17 | 17.11 |
| *Boana stellae* | 46.56 | 15.17 | 24.64 |
| *Boana stenocephala* | 28.3 | 7.7 | 14 |
| *Boana tepuiana* | 42.8 | 14.8 | 21.3 |
| *Boana wavrini* | 78 | 29.63 | 40.68 |
| *Boana xerophylla* | 57 | 19.8 | 32.2 |
| *Bokermannohyla ahenea* | 52.1 | 18.1 | 26.4 |
| *Bokermannohyla alvarengai* | 76 | 32 | 37.88 |
| *Bokermannohyla astartea* | 40.4 | 15.88 | 25.58 |
| *Bokermannohyla caramaschii* | 62.85 | 22.47 | 32.56 |
| *Bokermannohyla carvalhoi* | 61 | 23 | 33 |
| *Bokermannohyla circumdata* | 60 | 21.6 | 30 |
| *Bokermannohyla claresignata* | 61 | 23 | 33 |
| *Bokermannohyla clepsydra* | 53 | 18.75 | 27.75 |
| *Bokermannohyla gouveai* | 68 | 23.9 | 36 |
| *Bokermannohyla hylax* | 58.5 | 20.4 | 29.8 |
| *Bokermannohyla ibitiguara* | 40.48 | 15.18 | 20.72 |
| *Bokermannohyla ibitipoca* | 39.5 | 14.1 | 20 |
| *Bokermannohyla izecksohni* | 44.9 | 16.3 | 24.6 |
| *Bokermannohyla langei* | 66 | 23 | 32.92 |
| *Bokermannohyla lucianae* | 47.6 | 17.2 | 24.4 |
| *Bokermannohyla luctuosa* | 53.75 | 18.6 | 27.3 |
| *Bokermannohyla martinsi* | 56 | 21 | 28.66 |
| *Bokermannohyla nanuzae* | 41 | 20.5 | 20.5 |
| *Bokermannohyla pseudopseudis* | 43.5 | 102.3 | 23.68 |
| *Bokermannohyla ravida* | 42.1 | 16 | 22.1 |
| *Bokermannohyla sagarana* | 48.9 | 18.3 | 25.7 |
| *Bokermannohyla saxicola* | 50.1 | 17.69 | 25 |
| *Bokermannohyla sazimai* | 57.23 | 10.74 | 16.33 |
| *Bokermannohyla vulcaniae* | 46.75 | 17.1 | 24.98 |
| *Brachycephalus alipioi* | 15.3 | 6.8 | 6.8 |
| *Brachycephalus brunneus* | 10.2 | 3.9 | 3.3 |
| *Brachycephalus didactylus* | 10.2 | 4 | 4.3 |
| *Brachycephalus ephippium* | 19 | 7.5 | 7.5 |
| *Brachycephalus ferruginus* | 13.8 | 5.5 | 4.4 |
| *Brachycephalus hermogenesi* | 10 | 3.38 | 4.46 |
| *Brachycephalus izecksohni* | 11.1 | 4.5 | 3.8 |
| *Brachycephalus nodoterga* | 12.4 | 5.1 | 4.5 |
| *Brachycephalus pernix* | 14.9 | 5.94 | 4.15 |
| *Brachycephalus pombali* | 15 | 6 | 5.1 |
| *Brachycephalus vertebralis* | 12.9 | 5.3 | 4.5 |
| *Callimedusa atelopoides* | 42.5 | 16.3 | 16.3 |
| *Callimedusa baltea* | 63.5 | 21.8 | 29.4 |
| *Callimedusa tomopterna* | 55.5 | 19.63 | 24.26 |
| *Centrolene azulae* | 27.5 | 10 | 16.2 |
| *Centrolene bacatum* | 20.3 | 7.2 | 12.2 |
| *Centrolene medemi* | 26.1 | 10.3 | 18.7 |
| *Ceratophrys aurita* | 149 | 83.44 | 55.13 |
| *Ceratophrys cornuta* | 94 | 63 | 37.5 |
| *Ceratophrys cranwelli* | 83 | 48 | 28 |
| *Ceuthomantis aracamuni* | 18.2 | 6.2 | 11 |
| *Ceuthomantis cavernibardus* | 28.8 | 10.9 | 16.8 |
| *Ceuthomantis duellmani* | 24.3 | 8.6 | 14 |
| *Chiasmocleis alagoanus* | 26.8 | 7 | 10.2 |
| *Chiasmocleis albopunctata* | 32.6 | 8 | 10.3 |
| *Chiasmocleis anatipes* | 18.97 | 4.21 | 8.76 |
| *Chiasmocleis antenori* | 12.3 | 4.2 | 6 |
| *Chiasmocleis atlantica* | 30.6 | 8.1 | 12.3 |
| *Chiasmocleis avilapiresae* | 31.9 | 7.9 | 13.7 |
| *Chiasmocleis bassleri* | 25.6 | 6.6 | 11.9 |
| *Chiasmocleis capixaba* | 20.2 | 5.7 | 7.5 |
| *Chiasmocleis carvalhoi* | 20.2 | 5.6 | 7.9 |
| *Chiasmocleis centralis* | 23.6 | 6.8 | 8.72 |
| *Chiasmocleis cordeiroi* | 22.1 | 6.5 | 9 |
| *Chiasmocleis crucis* | 19.9 | 6.1 | 8.6 |
| *Chiasmocleis devriesi* | 42.2 | 12.5 | 15.4 |
| *Chiasmocleis gnoma* | 15.9 | 5.2 | 6.4 |
| *Chiasmocleis hudsoni* | 21.5 | 5.5 | 10.2 |
| *Chiasmocleis leucosticta* | 23.6 | 6.4 | 10.3 |
| *Chiasmocleis magnova* | 17.75 | 5.45 | 7.75 |
| *Chiasmocleis mantiqueira* | 21.5 | 6 | 10 |
| *Chiasmocleis mehelyi* | 19 | 5.5 | 6.4 |
| *Chiasmocleis sapiranga* | 23.5 | 6.4 | 9.5 |
| *Chiasmocleis schubarti* | 28.7 | 8 | 11.9 |
| *Chiasmocleis shudikarensis* | 26.7 | 6.9 | 11.9 |
| *Chiasmocleis tridactyla* | 12.4 | 4 | 5.1 |
| *Chiasmocleis ventrimaculata* | 25.5 | 5.4 | 10.5 |
| *Chimerella mariaelenae* | 19 | 7.4 | 10.8 |
| *Cochranella duidaeana* | 21.46 | 8.22 | 12.91 |
| *Cochranella erminea* | 23.4 | 7.6 | 13.6 |
| *Cochranella geijskesi* | 36.1 | 12.6 | 17 |
| *Cochranella resplendens* | 27.3 | 9.5 | 14.61 |
| *Cochranella riveroi* | 25 | 10.9 | 13.6 |
| *Colomascirtus armatus* | 64.3 | 21.8 | 34.2 |
| *Corythomantis greeningi* | 48 | 19.6 | 25.31 |
| *Crossodactylodes bokermanni* | 15.3 | 5.7 | 7.2 |
| *Crossodactylodes izecksohni* | 14.3 | 6 | 5.8 |
| *Crossodactylodes pintoi* | 17 | 7 | 7 |
| *Crossodactylus aeneus* | 31 | 10 | 14 |
| *Crossodactylus bokermanni* | 23.8 | 7.8 | 12.2 |
| *Crossodactylus caramaschii* | 24.5 | 7.94 | 12.69 |
| *Crossodactylus cyclospinus* | 29.4 | 8.8 | 13.3 |
| *Crossodactylus dantei* | 21.7 | 8 | 11.4 |
| *Crossodactylus dispar* | 26.9 | 9.5 | 12.4 |
| *Crossodactylus gaudichaudii* | 21.8 | 7.63 | 10.03 |
| *Crossodactylus grandis* | 34.5 | 12.7 | 15.8 |
| *Crossodactylus lutzorum* | 23.2 | 7.7 | 11.7 |
| *Crossodactylus schmidti* | 29 | 10 | 14 |
| *Crossodactylus trachystomus* | 24.2 | 7.8 | 11.8 |
| *Cruziohyla craspedopus* | 73 | 27.24 | 34.41 |
| *Ctenophryne barbatula* | 26.6 | 10.2 | 10 |
| *Ctenophryne carpish* | 33.7 | 12.6 | 11.7 |
| *Ctenophryne geayi* | 43.3 | 16.78 | 16.78 |
| *Cycloramphus acangatan* | 46.8 | 19.9 | 14.8 |
| *Cycloramphus asper* | 49.4 | 20.75 | 22.23 |
| *Cycloramphus bandeirensis* | 33.1 | 14.58 | 14.11 |
| *Cycloramphus bolitoglossus* | 39.8 | 17.11 | 13.53 |
| *Cycloramphus boraceiensis* | 55.4 | 23.18 | 24.79 |
| *Cycloramphus brasiliensis* | 68.9 | 28.94 | 33.07 |
| *Cycloramphus carvalhoi* | 58.8 | 27.1 | 18.7 |
| *Cycloramphus catarinensis* | 37.5 | 16 | 15.5 |
| *Cycloramphus cedrensis* | 40.5 | 17.3 | 18.7 |
| *Cycloramphus diringshofeni* | 30.6 | 12.24 | 15.3 |
| *Cycloramphus dubius* | 52.4 | 23.06 | 25.15 |
| *Cycloramphus duseni* | 38.4 | 15.74 | 18.43 |
| *Cycloramphus eleutherodactylus* | 46.2 | 19.5 | 23.32 |
| *Cycloramphus fuliginosus* | 54.7 | 23.52 | 24.07 |
| *Cycloramphus granulosus* | 37.8 | 15.74 | 15.88 |
| *Cycloramphus izecksohni* | 34.9 | 14.5 | 17.3 |
| *Cycloramphus juimirim* | 37.27 | 16.2 | 14.3 |
| *Cycloramphus lutzorum* | 45.1 | 19.2 | 22.1 |
| *Cycloramphus migueli* | 42.1 | 19.2 | 14 |
| *Cycloramphus mirandaribeiroi* | 60.2 | 27 | 29.7 |
| *Cycloramphus ohausi* | 36.2 | 15.56 | 15.56 |
| *Cycloramphus organensis* | 32.34 | 11.19 | 12.44 |
| *Cycloramphus rhyakonastes* | 42.2 | 17.8 | 21.6 |
| *Cycloramphus semipalmatus* | 45.5 | 20.55 | 21.51 |
| *Cycloramphus stejnegeri* | 49.6 | 20.83 | 16.86 |
| *Cycloramphus valae* | 36.2 | 13.4 | 16.3 |
| *Dasypops schirchi* | 52.28 | 9.95 | 19.86 |
| *Dendrobates leucomelas* | 33.8 | 10.39 | 14.75 |
| *Dendrobates nubeculosus* | 24.5 | 8 | 10.3 |
| *Dendrobates tinctorius* | 39 | 10.75 | 17.14 |
| *Dendrophryniscus berthalutzae* | 22 | 6.35 | 9.79 |
| *Dendrophryniscus brevipollicatus* | 21.2 | 7.52 | 11.5 |
| *Dendrophryniscus carvalhoi* | 18.5 | 5 | 8.51 |
| *Dendrophryniscus krausae* | 22.6 | 6.3 | 9.9 |
| *Dendrophryniscus leucomystax* | 25 | 7.5 | 10.3 |
| *Dendrophryniscus proboscideus* | 41.3 | 12.2 | 17.3 |
| *Dendrophryniscus stawiarskyi* | 22 | 6.71 | 9.69 |
| *Dendropsophus acreanus* | 33.25 | 10.75 | 16.83 |
| *Dendropsophus anataliasiasi* | 19.4 | 5.46 | 9.25 |
| *Dendropsophus anceps* | 42 | 14 | 22 |
| *Dendropsophus aperomeus* | 25 | 8.2 | 13.4 |
| *Dendropsophus araguaya* | 19.8 | 6 | 9.9 |
| *Dendropsophus berthalutzae* | 20 | 7 | 11.14 |
| *Dendropsophus bifurcus* | 25.7 | 8.68 | 13.98 |
| *Dendropsophus bipunctatus* | 24.5 | 8.5 | 12.5 |
| *Dendropsophus bokermanni* | 23.48 | 6.88 | 11.6 |
| *Dendropsophus branneri* | 18.5 | 6.06 | 10 |
| *Dendropsophus brevifrons* | 21.68 | 6.53 | 10.25 |
| *Dendropsophus cerradensis* | 18.9 | 6 | 9.6 |
| *Dendropsophus cruzi* | 22.9 | 6.6 | 11.9 |
| *Dendropsophus decipiens* | 19 | 7 | 10 |
| *Dendropsophus delarivai* | 26.6 | 9.1 | 14.6 |
| *Dendropsophus dutrai* | 38.1 | 10.7 | 18.93 |
| *Dendropsophus elegans* | 31.6 | 9.8 | 16.7 |
| *Dendropsophus elianeae* | 25.75 | 7.58 | 12.11 |
| *Dendropsophus gaucheri* | 19.2 | 6.73 | 11.94 |
| *Dendropsophus giesleri* | 25 | 8.72 | 13.18 |
| *Dendropsophus haddadi* | 22.6 | 7.5 | 11.34 |
| *Dendropsophus haraldschultzi* | 21.33 | 7 | 31.33 |
| *Dendropsophus jimi* | 21.5 | 6.35 | 10.45 |
| *Dendropsophus joannae* | 17.2 | 5.2 | 8.8 |
| *Dendropsophus juliani* | 20.7 | 7.5 | 10.3 |
| *Dendropsophus koechlini* | 28 | 8.8 | 13.6 |
| *Dendropsophus leali* | 20 | 6 | 12.58 |
| *Dendropsophus leucophyllatus* | 27 | 9 | 14 |
| *Dendropsophus limai* | 19 | 6.5 | 9.36 |
| *Dendropsophus marmoratus* | 35.5 | 11.7 | 21.5 |
| *Dendropsophus mathiassoni* | 21.4 | 6.6 | 10.2 |
| *Dendropsophus melanargyreus* | 37 | 11 | 18 |
| *Dendropsophus meridianus* | 24 | 8.13 | 13.39 |
| *Dendropsophus microcephalus* | 22.8 | 29.4 | 52.9 |
| *Dendropsophus microps* | 22.5 | 8.5 | 14.36 |
| *Dendropsophus minimus* | 23.26 | 8.5 | 14.03 |
| *Dendropsophus minusculus* | 20 | 6.5 | 9.7 |
| *Dendropsophus minutus* | 21.1 | 6.75 | 10.97 |
| *Dendropsophus miyatai* | 17.34 | 5.93 | 9.51 |
| *Dendropsophus nahdereri* | 41 | 14.73 | 21.18 |
| *Dendropsophus nanus* | 20 | 6 | 9.5 |
| *Dendropsophus novaisi* | 39 | 11.5 | 19.18 |
| *Dendropsophus oliveirai* | 17.8 | 5.8 | 8.16 |
| *Dendropsophus parviceps* | 23.45 | 6.75 | 11.87 |
| *Dendropsophus pauiniensis* | 20.2 | 6.8 | 9.9 |
| *Dendropsophus pseudomeridianus* | 21.8 | 6.8 | 10.7 |
| *Dendropsophus reichlei* | 21.5 | 7.6 | 11.6 |
| *Dendropsophus rhea* | 19 | 5.9 | 9.46 |
| *Dendropsophus rhodopeplus* | 27.3 | 9.28 | 14.5 |
| *Dendropsophus riveroi* | 20 | 6.4 | 10.4 |
| *Dendropsophus rossalleni* | 22.8 | 8.3 | 12.53 |
| *Dendropsophus rubicundulus* | 22 | 7 | 10 |
| *Dendropsophus ruschii* | 27.9 | 8.7 | 13.8 |
| *Dendropsophus sanborni* | 20 | 5.5 | 10 |
| *Dendropsophus sarayacuensis* | 25.6 | 9.1 | 14.6 |
| *Dendropsophus schubarti* | 18.66 | 6.09 | 11.19 |
| *Dendropsophus seniculus* | 37.7 | 12.44 | 18.85 |
| *Dendropsophus soaresi* | 30.9 | 9 | 13.5 |
| *Dendropsophus studerae* | 28.2 | 9.4 | 15.4 |
| *Dendropsophus timbeba* | 22.5 | 7.5 | 11.3 |
| *Dendropsophus tintinnabulum* | 20 | 6.27 | 11.23 |
| *Dendropsophus triangulum* | 27.6 | 9.61 | 14.96 |
| *Dendropsophus tritaeniatus* | 22 | 6.5 | 13.49 |
| *Dendropsophus walfordi* | 19.5 | 6.5 | 9.96 |
| *Dendropsophus werneri* | 20.5 | 6.5 | 10 |
| *Dendropsophus xapuriensis* | 16.69 | 5.96 | 9.74 |
| *Dermatonotus muelleri* | 54.3 | 17.85 | 21.7 |
| *Dryaderces inframaculata* | 51.9 | 16.67 | 27.18 |
| *Dryaderces pearsoni* | 54.7 | 18.93 | 28.01 |
| *Edalorhina nasuta* | 38 | 13.78 | 17.35 |
| *Edalorhina perezi* | 22 | 7.5 | 10 |
| *Elachistocleis bicolor* | 42.5 | 11.82 | 15.69 |
| *Elachistocleis bumbameuboi* | 37.4 | 8.6 | 12.9 |
| *Elachistocleis carvalhoi* | 35.2 | 8.4 | 13.2 |
| *Elachistocleis erythrogaster* | 35.46 | 7.2 | 11.68 |
| *Elachistocleis helianneae* | 31.7 | 7.3 | 11.8 |
| *Elachistocleis matogrosso* | 30.6 | 7.1 | 10.65 |
| *Elachistocleis ovalis* | 36 | 9 | 12.5 |
| *Elachistocleis piauiensis* | 22.5 | 5.8 | 8.2 |
| *Elachistocleis surinamensis* | 37.44 | 9.71 | 13.27 |
| *Elachistocleis surumu* | 25.6 | 6.65 | 8.9 |
| *Eleutherodactylus johnstonei* | 29.1 | 10.5 | 11.3 |
| *Engystomops freibergi* | 27.5 | 8 | 14 |
| *Engystomops petersi* | 30 | 10 | 14.85 |
| *Engystomops pustulosus* | 30 | 10 | 12 |
| *Espadarana durrellorum* | 25.7 | 8.7 | 14.6 |
| *Euparkerella brasiliensis* | 20.5 | 8.5 | 8 |
| *Euparkerella cochranae* | 15 | 6 | 6.5 |
| *Euparkerella robusta* | 17 | 6.5 | 6.7 |
| *Euparkerella tridactyla* | 22 | 9.5 | 8 |
| *Excidobates captivus* | 14.16 | 4.68 | 6.57 |
| *Fritziana fissilis* | 22.9 | 8.24 | 12.6 |
| *Fritziana goeldii* | 32.5 | 11 | 15.5 |
| *Fritziana ohausi* | 32.1 | 10.59 | 15.41 |
| *Frostius erythrophthalmus* | 23.4 | 8.3 | 9.2 |
| *Frostius pernambucensis* | 20.17 | 7.05 | 7.25 |
| *Gastrotheca albolineata* | 55 | 21.6 | 25.4 |
| *Gastrotheca andaquiensis* | 73.7 | 28.35 | 47.5 |
| *Gastrotheca antoniiochoai* | 27.8 | 8.9 | 15.1 |
| *Gastrotheca carinaceps* | 79.7 | 29 | 44 |
| *Gastrotheca ernestoi* | 63 | 23 | 33.48 |
| *Gastrotheca excubitor* | 37.1 | 14.02 | 18.92 |
| *Gastrotheca fissipes* | 67.2 | 25.9 | 30.8 |
| *Gastrotheca flamma* | 55.3 | 20.3 | 27.9 |
| *Gastrotheca fulvorufa* | 68.8 | 25.54 | 38.14 |
| *Gastrotheca longipes* | 55.7 | 21.4 | 35.1 |
| *Gastrotheca marsupiata* | 46.5 | 16.38 | 24.82 |
| *Gastrotheca microdiscus* | 70 | 26 | 39 |
| *Gastrotheca nicefori* | 65.3 | 25.7 | 36.4 |
| *Gastrotheca ochoai* | 33.4 | 11.72 | 17.6 |
| *Gastrotheca stictopleura* | 68.3 | 23.1 | 34.1 |
| *Gastrotheca testudinea* | 76.67 | 27.5 | 36.5 |
| *Gastrotheca weinlandii* | 90 | 36 | 48.6 |
| *Haddadus binotatus* | 57.4 | 22.39 | 32.14 |
| *Hamptophryne alios* | 49.5 | 20.5 | 16.6 |
| *Hamptophryne boliviana* | 39 | 14.61 | 13.4 |
| *Hemiphractus bubalus* | 60.7 | 27 | 33.7 |
| *Hemiphractus helioi* | 52.7 | 27 | 27.3 |
| *Hemiphractus johnsoni* | 45.5 | 23.3 | 26.5 |
| *Hemiphractus proboscideus* | 44.4 | 25.5 | 25 |
| *Hemiphractus scutatus* | 48 | 23.55 | 22 |
| *Holoaden bradei* | 30.8 | 11.7 | 11 |
| *Holoaden luederwaldti* | 39.6 | 16.2 | 15.9 |
| *Holoaden pholeter* | 44.6 | 16.3 | 19 |
| *Hyalinobatrachium cappellei* | 23 | 8.7 | 13.2 |
| *Hyalinobatrachium fleischmanni* | 19.2 | 7 | 10.8 |
| *Hyalinobatrachium iaspidiense* | 22.7 | 8.8 | 12.5 |
| *Hyalinobatrachium ruedai* | 21.2 | 7.82 | 11.8 |
| *Hyalinobatrachium taylori* | 20.84 | 7.34 | 11.25 |
| *Hydrolaetare dantasi* | 84.3 | 36.4 | 35.2 |
| *Hydrolaetare schmidti* | 82 | 34.6 | 36.3 |
| *Hylodes amnicola* | 28.8 | 8.9 | 15.4 |
| *Hylodes asper* | 45.5 | 15.02 | 25.03 |
| *Hylodes babax* | 30.6 | 9.7 | 17.2 |
| *Hylodes cardosoi* | 42.4 | 14 | 23.2 |
| *Hylodes charadranaetes* | 34.4 | 10.4 | 17.3 |
| *Hylodes dactylocinus* | 25.4 | 8.1 | 12.7 |
| *Hylodes glaber* | 38.8 | 12.5 | 21.7 |
| *Hylodes heyeri* | 45 | 13.5 | 23.4 |
| *Hylodes lateristrigatus* | 39 | 12.5 | 21 |
| *Hylodes magalhaesi* | 33 | 10.5 | 19.2 |
| *Hylodes meridionalis* | 41.7 | 13.6 | 21.2 |
| *Hylodes mertensi* | 51.5 | 17.6 | 28.1 |
| *Hylodes nasus* | 38 | 13 | 20 |
| *Hylodes ornatus* | 24.9 | 7.5 | 13.1 |
| *Hylodes otavioi* | 32.46 | 9.8 | 18.56 |
| *Hylodes perplicatus* | 38.6 | 13 | 22.7 |
| *Hylodes phyllodes* | 31.8 | 9.54 | 13.99 |
| *Hylodes pipilans* | 25.1 | 7.8 | 13.8 |
| *Hylodes regius* | 35.6 | 11.6 | 19.1 |
| *Hylodes sazimai* | 27.6 | 9.2 | 14.4 |
| *Hylodes uai* | 32.7 | 11.2 | 17.5 |
| *Hylodes vanzolinii* | 29 | 9.5 | 16.9 |
| *Hylomantis aspera* | 41.7 | 15.5 | 20.4 |
| *Hylomantis granulosa* | 37.4 | 14.5 | 17.2 |
| *Hyloscirtus albopunctulatus* | 41.5 | 14 | 21.7 |
| *Hyloscirtus phyllognathus* | 36.87 | 12.13 | 18.25 |
| *Hyloscirtus torrenticola* | 34.9 | 12.11 | 17.24 |
| *Hyloxalus azureiventris* | 27 | 9.11 | 13.04 |
| *Hyloxalus bocagei* | 27.3 | 9.5 | 12.8 |
| *Hyloxalus cevallosi* | 18.2 | 6.2 | 10.1 |
| *Hyloxalus chlorocraspedus* | 28.2 | 8.9 | 14 |
| *Hyloxalus craspedoceps* | 19.5 | 7 | 8.8 |
| *Hyloxalus eleutherodactylus* | 22.7 | 7.3 | 10.3 |
| *Hyloxalus exasperatus* | 20.5 | 7 | 9.4 |
| *Hyloxalus faciopunctulatus* | 23.83 | 8.97 | 10.87 |
| *Hyloxalus idiomelus* | 25.3 | 8.1 | 12.2 |
| *Hyloxalus mittermeieri* | 25.03 | 8.48 | 12.32 |
| *Hyloxalus nexipus* | 21.4 | 7.58 | 10.79 |
| *Hyloxalus patitae* | 22.6 | 7.91 | 11.07 |
| *Hyloxalus pulchellus* | 20.4 | 6.6 | 9.2 |
| *Hyloxalus sauli* | 22.41 | 8.52 | 11.47 |
| *Hyloxalus shuar* | 26.5 | 11.1 | 13.2 |
| *Hyloxalus sordidatus* | 33 | 11.2 | 15.5 |
| *Hypodactylus dolops* | 57.6 | 24.2 | 34.1 |
| *Hypodactylus nigrovittatus* | 21.1 | 8.27 | 9.58 |
| *Ischnocnema bolbodactyla* | 20.4 | 6.5 | 9.8 |
| *Ischnocnema epipeda* | 20.5 | 6.9 | 10.9 |
| *Ischnocnema erythromera* | 23.6 | 8.5 | 14.9 |
| *Ischnocnema gehrti* | 21.54 | 7.35 | 11.2 |
| *Ischnocnema gualteri* | 29.1 | 15.68 | 19.73 |
| *Ischnocnema guentheri* | 36.1 | 13 | 23.1 |
| *Ischnocnema henselii* | 33.4 | 11.8 | 20.7 |
| *Ischnocnema hoehnei* | 29.4 | 9.41 | 18.82 |
| *Ischnocnema holti* | 25.6 | 9.2 | 12.5 |
| *Ischnocnema izecksohni* | 43.5 | 16 | 25.8 |
| *Ischnocnema juipoca* | 25.1 | 9.4 | 13.7 |
| *Ischnocnema lactea* | 33 | 11 | 15 |
| *Ischnocnema manezinho* | 33.06 | 12.59 | 19.31 |
| *Ischnocnema nasuta* | 33 | 11 | 20 |
| *Ischnocnema nigriventris* | 19 | 6.84 | 9.12 |
| *Ischnocnema octavioi* | 31.25 | 12 | 17.57 |
| *Ischnocnema oea* | 17.7 | 6 | 11.1 |
| *Ischnocnema paranaensis* | 17.7 | 6.1 | 8.6 |
| *Ischnocnema parva* | 19.7 | 7.49 | 9.26 |
| *Ischnocnema penaxavantinho* | 20.3 | 7.2 | 11 |
| *Ischnocnema pusilla* | 16 | 6.5 | 11.62 |
| *Ischnocnema randorum* | 16.6 | 5.64 | 7.97 |
| *Ischnocnema sambaqui* | 36.89 | 13.96 | 19.16 |
| *Ischnocnema spanios* | 21.4 | 7.28 | 11.13 |
| *Ischnocnema venancioi* | 25.5 | 7.5 | 12 |
| *Ischnocnema verrucosa* | 22.1 | 9.3 | 12 |
| *Itapotihyla langsdorffii* | 74.9 | 21.72 | 40.45 |
| *Julianus pinimus* | 29 | 9.6 | 13.3 |
| *Julianus uruguayus* | 24.6 | 8.3 | 12.8 |
| *Leptodactylus bolivianus* | 89.3 | 30.9 | 43.8 |
| *Leptodactylus bufonius* | 53.6 | 18.28 | 21.33 |
| *Leptodactylus caatingae* | 35.3 | 12.7 | 14.1 |
| *Leptodactylus camaquara* | 32 | 15 | 11.04 |
| *Leptodactylus chaquensis* | 77 | 28.33 | 35.5 |
| *Leptodactylus colombiensis* | 46.3 | 15.5 | 21.3 |
| *Leptodactylus cunicularius* | 40 | 15 | 22 |
| *Leptodactylus cupreus* | 52.4 | 18 | 26.1 |
| *Leptodactylus didymus* | 46.7 | 15.8 | 25.1 |
| *Leptodactylus diedrus* | 34.3 | 12.4 | 16.9 |
| *Leptodactylus discodactylus* | 35 | 13.65 | 17.15 |
| *Leptodactylus elenae* | 43.5 | 14.3 | 20.6 |
| *Leptodactylus flavopictus* | 131.6 | 55.27 | 61.85 |
| *Leptodactylus fragilis* | 34.2 | 11.39 | 15.6 |
| *Leptodactylus furnarius* | 35.4 | 11.6 | 11.98 |
| *Leptodactylus fuscus* | 45.5 | 15.93 | 23.66 |
| *Leptodactylus gracilis* | 43 | 13.63 | 24.64 |
| *Leptodactylus griseigularis* | 46.6 | 15.84 | 21.44 |
| *Leptodactylus hylodes* | 25.3 | 8.7 | 12.1 |
| *Leptodactylus jolyi* | 45.4 | 17 | 34.05 |
| *Leptodactylus knudseni* | 132 | 48.84 | 54.12 |
| *Leptodactylus labyrinthicus* | 141.8 | 56.72 | 62.39 |
| *Leptodactylus latinasus* | 34 | 11.5 | 14 |
| *Leptodactylus latrans* | 96.8 | 32.91 | 46.46 |
| *Leptodactylus lauramiriamae* | 31.2 | 11.6 | 11.5 |
| *Leptodactylus leptodactyloides* | 46.3 | 15.74 | 21.76 |
| *Leptodactylus lithonaetes* | 71.4 | 27.4 | 31.5 |
| *Leptodactylus longirostris* | 41.8 | 13.96 | 22.03 |
| *Leptodactylus myersi* | 109.4 | 41.38 | 43.76 |
| *Leptodactylus mystaceus* | 43.6 | 14.91 | 22.54 |
| *Leptodactylus mystacinus* | 57.3 | 20.06 | 24.07 |
| *Leptodactylus natalensis* | 39.9 | 13.57 | 17.96 |
| *Leptodactylus notoaktites* | 56.1 | 18.4 | 31.5 |
| *Leptodactylus paraensis* | 128.7 | 49.9 | 54 |
| *Leptodactylus pentadactylus* | 148.1 | 57.76 | 66.65 |
| *Leptodactylus petersii* | 39.1 | 13.69 | 17.2 |
| *Leptodactylus plaumanni* | 41.93 | 14.17 | 25.53 |
| *Leptodactylus podicipinus* | 38.8 | 12.8 | 15.91 |
| *Leptodactylus pustulatus* | 51 | 17.85 | 22.44 |
| *Leptodactylus rhodomystax* | 76.5 | 29.07 | 33.66 |
| *Leptodactylus rhodonotus* | 75 | 27.75 | 31.5 |
| *Leptodactylus rhodostima* | 69.68 | 26.2 | 31.09 |
| *Leptodactylus riveroi* | 62.8 | 23.2 | 29.9 |
| *Leptodactylus rugosus* | 68.2 | 26.6 | 30.69 |
| *Leptodactylus sabanensis* | 51 | 17.34 | 24.99 |
| *Leptodactylus sertanejo* | 54.3 | 14.8 | 35.1 |
| *Leptodactylus spixi* | 43 | 14.5 | 21.5 |
| *Leptodactylus stenodema* | 91.1 | 32.8 | 35.53 |
| *Leptodactylus syphax* | 79.7 | 29.1 | 30 |
| *Leptodactylus tapiti* | 32 | 12 | 8.68 |
| *Leptodactylus troglodytes* | 49.9 | 16.92 | 19.81 |
| *Leptodactylus validus* | 36.9 | 11.81 | 16.97 |
| *Leptodactylus vastus* | 151.1 | 60.14 | 62.4 |
| *Leptodactylus viridis* | 66.4 | 23.8 | 29.7 |
| *Leptodactylus wagneri* | 65.5 | 22.27 | 33.41 |
| *Leucostethus argyrogaster* | 22.1 | 6.87 | 9.81 |
| *Leucostethus fugax* | 20.1 | 5.99 | 9.05 |
| *Limnomedusa macroglossa* | 48 | 19 | 33 |
| *Lithobates palmipes* | 75 | 28.5 | 38 |
| *Lithodytes lineatus* | 54 | 17.5 | 25 |
| *Lysapsus bolivianus* | 18 | 7 | 12 |
| *Lysapsus caraya* | 16.5 | 3 | 11.5 |
| *Lysapsus laevis* | 21 | 6.43 | 13.4 |
| *Lysapsus limellum* | 17 | 6 | 12 |
| *Macrogenioglottus alipioi* | 72 | 50 | 32 |
| *Megaelosia apuana* | 92.2 | 38.6 | 45.1 |
| *Megaelosia bocainensis* | 66.7 | 27 | 32.6 |
| *Megaelosia boticariana* | 74.9 | 34.45 | 39.7 |
| *Megaelosia goeldii* | 89.98 | 34.83 | 45.15 |
| *Megaelosia jordanensis* | 47 | 20.6 | 23 |
| *Megaelosia lutzae* | 90 | 35.1 | 40.5 |
| *Megaelosia massarti* | 115.43 | 47.9 | 56.1 |
| *Melanophryniscus admirabilis* | 35.53 | 10.4 | 13.04 |
| *Melanophryniscus alipioi* | 21.06 | 6.81 | 7.58 |
| *Melanophryniscus atroluteus* | 21 | 5.08 | 7.81 |
| *Melanophryniscus cambaraensis* | 34.2 | 10.4 | 14.2 |
| *Melanophryniscus dorsalis* | 23.95 | 7.55 | 8.35 |
| *Melanophryniscus fulvoguttatus* | 27.1 | 7.9 | 8.7 |
| *Melanophryniscus macrogranulosus* | 33.58 | 9.57 | 11.79 |
| *Melanophryniscus moreirae* | 27.4 | 7.42 | 9.22 |
| *Melanophryniscus peritus* | 39.3 | 9.3 | 11.7 |
| *Melanophryniscus simplex* | 28.6 | 8.9 | 10.1 |
| *Melanophryniscus spectabilis* | 33.6 | 10.4 | 11.7 |
| *Melanophryniscus tumifrons* | 30 | 8.8 | 11.5 |
| *Myersiella microps* | 15 | 4.65 | 6.15 |
| *Myersiohyla aromatica* | 44.5 | 16 | 24.4 |
| *Myersiohyla kanaima* | 47.6 | 16.1 | 24.2 |
| *Myersiohyla loveridgei* | 42 | 15 | 22 |
| *Noblella lochites* | 14.9 | 5.2 | 7 |
| *Noblella myrmecoides* | 13.6 | 4.75 | 6.4 |
| *Nyctimantis rugiceps* | 61.3 | 22.31 | 32.18 |
| *Nymphargus bejaranoi* | 24.4 | 8.18 | 13.81 |
| *Nymphargus laurae* | 19.9 | 7.4 | 11.7 |
| *Nymphargus mariae* | 30 | 10.4 | 16.6 |
| *Nymphargus mixomaculatus* | 26.3 | 8.8 | 14.9 |
| *Nymphargus ocellatus* | 29 | 10.4 | 18.15 |
| *Nymphargus phenax* | 22.1 | 7.82 | 12.8 |
| *Nymphargus posadae* | 31.2 | 10.23 | 19.83 |
| *Nymphargus siren* | 20.7 | 7.18 | 11.86 |
| *Nymphargus truebae* | 23.3 | 8.46 | 15.56 |
| *Odontophrynus americanus* | 44.6 | 18.91 | 11.73 |
| *Odontophrynus carvalhoi* | 67.9 | 29.2 | 23.7 |
| *Odontophrynus cultripes* | 58.6 | 22.85 | 16.58 |
| *Odontophrynus salvatori* | 27.3 | 12.25 | 10.6 |
| *Ololygon agilis* | 16 | 6 | 9 |
| *Ololygon albicans* | 38 | 12.5 | 19.16 |
| *Ololygon angrensis* | 28.5 | 9.51 | 14.14 |
| *Ololygon arduous* | 26.2 | 8.38 | 13.67 |
| *Ololygon argyreornata* | 20.48 | 6.68 | 11.05 |
| *Ololygon ariadne* | 43 | 15 | 23.83 |
| *Ololygon atrata* | 19.4 | 7.2 | 8.9 |
| *Ololygon belloni* | 28.4 | 10.4 | 15.3 |
| *Ololygon berthae* | 18.1 | 6 | 9 |
| *Ololygon brieni* | 34.5 | 11.39 | 17.6 |
| *Ololygon canastrensis* | 28.78 | 8.93 | 16.9 |
| *Ololygon carnevallii* | 24.5 | 8.3 | 13.6 |
| *Ololygon catharinae* | 45 | 15.3 | 22.39 |
| *Ololygon centralis* | 19.74 | 6.53 | 10.73 |
| *Ololygon faivovichi* | 19.9 | 6.3 | 10.6 |
| *Ololygon flavoguttata* | 42 | 15 | 21 |
| *Ololygon heyeri* | 36.6 | 12.41 | 19.38 |
| *Ololygon hiemalis* | 28.17 | 9.39 | 14 |
| *Ololygon humilis* | 31.5 | 9.91 | 15.77 |
| *Ololygon insperata* | 23.8 | 8.5 | 13.1 |
| *Ololygon jureia* | 29.3 | 9.5 | 16.1 |
| *Ololygon kautskyi* | 28.6 | 9.5 | 15.7 |
| *Ololygon littoralis* | 28.9 | 9 | 16.5 |
| *Ololygon littoreus* | 19.3 | 4.95 | 9.65 |
| *Ololygon longilinea* | 48 | 17 | 25 |
| *Ololygon luizotavioi* | 24.1 | 7.7 | 12.2 |
| *Ololygon machadoi* | 24.3 | 9 | 10 |
| *Ololygon melloi* | 18.7 | 6.23 | 9.16 |
| *Ololygon obtriangulatus* | 24.5 | 7.46 | 12.35 |
| *Ololygon perpusilla* | 19.8 | 6.53 | 10.1 |
| *Ololygon ranki* | 27.15 | 9.25 | 14.95 |
| *Ololygon rizibilis* | 34 | 10 | 16.79 |
| *Ololygon trapicheiroi* | 40 | 13.75 | 21.08 |
| *Ololygon v.signata* | 25.7 | 7.7 | 12.8 |
| *Oreobates crepitans* | 32 | 11.5 | 16.07 |
| *Oreobates cruralis* | 29.3 | 10.1 | 14.5 |
| *Oreobates heterodactylus* | 26.77 | 9.42 | 13.4 |
| *Oreobates lehri* | 24.4 | 9.3 | 15.7 |
| *Oreobates quixensis* | 58 | 25 | 32.66 |
| *Oreobates saxatilis* | 51.5 | 20.3 | 31.4 |
| *Oreophrynella cryptica* | 20.8 | 7.1 | 7.7 |
| *Oreophrynella dendronastes* | 33.9 | 11.8 | 13.3 |
| *Oreophrynella huberi* | 19 | 7 | 7 |
| *Oreophrynella macconnelli* | 22 | 7.54 | 8.25 |
| *Osteocephalus alboguttatus* | 45 | 15.55 | 24.13 |
| *Osteocephalus buckleyi* | 61.7 | 21.47 | 34.12 |
| *Osteocephalus cabrerai* | 52.7 | 19.2 | 31.8 |
| *Osteocephalus castaneicola* | 57.6 | 18.4 | 31.9 |
| *Osteocephalus deridens* | 47.8 | 16.18 | 26.92 |
| *Osteocephalus fuscifacies* | 53.2 | 19.7 | 27.5 |
| *Osteocephalus heyeri* | 42.8 | 14.3 | 21.9 |
| *Osteocephalus leoniae* | 40.1 | 13.8 | 21.5 |
| *Osteocephalus leprieurii* | 56.52 | 19.05 | 30.9 |
| *Osteocephalus mimeticus* | 34 | 12 | 23 |
| *Osteocephalus mutabor* | 48.3 | 18.1 | 25.9 |
| *Osteocephalus oophagus* | 52.16 | 16.25 | 28.75 |
| *Osteocephalus planiceps* | 63.2 | 20.4 | 36.6 |
| *Osteocephalus subtilis* | 37.23 | 13.23 | 20.37 |
| *Osteocephalus taurinus* | 82.5 | 26.41 | 43.6 |
| *Osteocephalus verruciger* | 64.5 | 23.09 | 35.15 |
| *Osteocephalus yasuni* | 52.7 | 17.8 | 31 |
| *Otophryne pyburni* | 56.3 | 18.7 | 18.6 |
| *Otophryne robusta* | 51.7 | 18.3 | 11.8 |
| *Otophryne steyermarki* | 35.1 | 11.6 | 14.6 |
| *Paratelmatobius cardosoi* | 21.75 | 8.14 | 9.8 |
| *Paratelmatobius gaigeae* | 16.7 | 5.5 | 7.08 |
| *Paratelmatobius lutzii* | 23 | 8 | 9 |
| *Paratelmatobius mantiqueira* | 16.8 | 6.31 | 6.81 |
| *Paratelmatobius poecilogaster* | 26.05 | 10.1 | 11.9 |
| *Phasmahyla cochranae* | 31.25 | 11.25 | 15.31 |
| *Phasmahyla exilis* | 34.5 | 11 | 18.5 |
| *Phasmahyla guttata* | 35 | 11 | 17 |
| *Phasmahyla jandaia* | 30.4 | 11.5 | 15.52 |
| *Phasmahyla spectabilis* | 45.6 | 15.1 | 22.2 |
| *Phasmahyla timbo* | 35.2 | 12.8 | 17.3 |
| *Phrynomedusa appendiculata* | 37.4 | 12.71 | 18.33 |
| *Phrynomedusa bokermanni* | 46 | 16 | 23 |
| *Phrynomedusa marginata* | 28 | 11 | 13 |
| *Phrynomedusa vanzolinii* | 36 | 12.9 | 17.8 |
| *Phrynopus bracki* | 19.8 | 7.81 | 7.4 |
| *Phyllodytes acuminatus* | 24.5 | 11 | 12.29 |
| *Phyllodytes edelmoi* | 26.2 | 9.5 | 13.8 |
| *Phyllodytes gyrinaethes* | 26 | 9.7 | 13 |
| *Phyllodytes kautskyi* | 38 | 13.9 | 18.6 |
| *Phyllodytes luteolus* | 23 | 8.15 | 10.94 |
| *Phyllodytes maculosus* | 49.5 | 18.6 | 22.1 |
| *Phyllodytes melanomystax* | 24.5 | 8.9 | 11.7 |
| *Phyllodytes tuberculosus* | 23 | 8.6 | 11.2 |
| *Phyllodytes wuchereri* | 26.66 | 9.93 | 12.57 |
| *Phyllomedusa bahiana* | 75 | 23 | 27 |
| *Phyllomedusa bicolor* | 110.4 | 43.7 | 46.1 |
| *Phyllomedusa boliviana* | 62.6 | 21.8 | 32.4 |
| *Phyllomedusa burmeisteri* | 35 | 11 | 17 |
| *Phyllomedusa camba* | 81.46 | 27.29 | 35.23 |
| *Phyllomedusa coelestis* | 64.8 | 22.8 | 31.4 |
| *Phyllomedusa distincta* | 70 | 21 | 28 |
| *Phyllomedusa iheringii* | 74 | 24 | 26 |
| *Phyllomedusa sauvagii* | 70 | 23.16 | 29.18 |
| *Phyllomedusa tarsius* | 95.5 | 32.5 | 45.1 |
| *Phyllomedusa tetraploidea* | 59.05 | 19.5 | 24.23 |
| *Phyllomedusa vaillantii* | 44 | 16.43 | 18.65 |
| *Physalaemus aguirrei* | 30 | 9.5 | 14.09 |
| *Physalaemus albifrons* | 34 | 11.05 | 16.63 |
| *Physalaemus albonotatus* | 30.2 | 9.61 | 13.63 |
| *Physalaemus angrensis* | 19.73 | 6.2 | 9.88 |
| *Physalaemus atlanticus* | 22.4 | 7.1 | 10.7 |
| *Physalaemus barrioi* | 26.67 | 8.56 | 13.07 |
| *Physalaemus biligonigerus* | 37.5 | 12.61 | 17.04 |
| *Physalaemus bokermanni* | 16.22 | 6 | 8.02 |
| *Physalaemus caete* | 24.54 | 9.1 | 12.31 |
| *Physalaemus camacan* | 23.3 | 7.4 | 11.1 |
| *Physalaemus centralis* | 43 | 13 | 18.4 |
| *Physalaemus cicada* | 27.5 | 8 | 12.81 |
| *Physalaemus crombiei* | 21.8 | 8.5 | 10.46 |
| *Physalaemus cuvieri* | 30.6 | 9.18 | 13.16 |
| *Physalaemus deimaticus* | 24.3 | 7.9 | 10.25 |
| *Physalaemus ephippifer* | 28.5 | 8.1 | 15.1 |
| *Physalaemus erikae* | 23.1 | 7.4 | 11.6 |
| *Physalaemus erythros* | 26.6 | 8.1 | 10.8 |
| *Physalaemus evangelistai* | 21.5 | 6 | 10.47 |
| *Physalaemus fischeri* | 33 | 9.3 | 13.6 |
| *Physalaemus gracilis* | 30 | 9.93 | 14.34 |
| *Physalaemus henselii* | 19 | 5.12 | 8.5 |
| *Physalaemus insperatus* | 26.2 | 7.5 | 12.23 |
| *Physalaemus irroratus* | 22.9 | 7.6 | 10.2 |
| *Physalaemus jordanensis* | 24 | 7.2 | 11.66 |
| *Physalaemus kroyeri* | 32 | 10.29 | 14.51 |
| *Physalaemus lisei* | 25.04 | 7.21 | 13.1 |
| *Physalaemus maculiventris* | 22.33 | 6.52 | 10.95 |
| *Physalaemus marmoratus* | 41.8 | 12.5 | 16.2 |
| *Physalaemus maximus* | 46.3 | 14.1 | 21.9 |
| *Physalaemus moreirae* | 25.4 | 8.8 | 11.9 |
| *Physalaemus nanus* | 18 | 3.53 | 6.85 |
| *Physalaemus nattereri* | 40.65 | 13.69 | 19.71 |
| *Physalaemus obtectus* | 26 | 8 | 12.12 |
| *Physalaemus olfersii* | 31.9 | 9.89 | 14.67 |
| *Physalaemus riograndensis* | 18.6 | 5.84 | 8.91 |
| *Physalaemus rupestris* | 17.8 | 5.2 | 7.1 |
| *Physalaemus signifer* | 21.5 | 6.5 | 10.5 |
| *Physalaemus soaresi* | 24 | 6.9 | 10.5 |
| *Physalaemus spiniger* | 21.1 | 6.3 | 10.2 |
| *Phyzelaphryne miriamae* | 19.6 | 7 | 8.9 |
| *Pipa arrabali* | 40 | 10.5 | 18.5 |
| *Pipa aspera* | 45 | 11 | 20.6 |
| *Pipa carvalhoi* | 57.7 | 15.1 | 23.4 |
| *Pipa pipa* | 134 | 54 | 53.5 |
| *Pipa snethlageae* | 89 | 27.5 | 31 |
| *Pithecopus ayeaye* | 39.17 | 12.5 | 15.67 |
| *Pithecopus azureus* | 43.1 | 12.1 | 16.6 |
| *Pithecopus centralis* | 42 | 14.5 | 12.84 |
| *Pithecopus hypochondrialis* | 35 | 11.8 | 16.3 |
| *Pithecopus megacephalus* | 43.7 | 14.4 | 17 |
| *Pithecopus nordestinus* | 41.1 | 12.1 | 16.5 |
| *Pithecopus oreades* | 36.48 | 12.21 | 14.54 |
| *Pithecopus palliata* | 43.6 | 14.26 | 18.6 |
| *Pithecopus rohdei* | 42 | 13.77 | 19.34 |
| *Pleurodema bibroni* | 31 | 13 | 14 |
| *Pleurodema brachyops* | 36 | 14.5 | 15 |
| *Pleurodema diplolister* | 33.8 | 13.3 | 13.6 |
| *Pleurodema marmoratum* | 31.8 | 10.5 | 10.9 |
| *Pristimantis aaptus* | 22.9 | 8.2 | 12.5 |
| *Pristimantis acuminatus* | 24 | 9.04 | 13.09 |
| *Pristimantis adiastolus* | 28 | 8.9 | 17.3 |
| *Pristimantis albertus* | 20.2 | 7 | 10.45 |
| *Pristimantis altamazonicus* | 27.9 | 10.35 | 13.62 |
| *Pristimantis altamnis* | 27.6 | 10.8 | 15.2 |
| *Pristimantis ardalonychus* | 27.4 | 10.4 | 13.7 |
| *Pristimantis aureolineatus* | 27.5 | 11.1 | 14.5 |
| *Pristimantis aureoventris* | 26.8 | 10.4 | 14.9 |
| *Pristimantis avius* | 33 | 13.1 | 17.9 |
| *Pristimantis bearsei* | 38.4 | 15.7 | 19.3 |
| *Pristimantis bromeliaceus* | 40.2 | 14.1 | 19.5 |
| *Pristimantis buccinator* | 28.2 | 9.6 | 16.1 |
| *Pristimantis cantitans* | 33 | 13.3 | 17.8 |
| *Pristimantis carvalhoi* | 17 | 6 | 10 |
| *Pristimantis chiastonotus* | 38.4 | 12.3 | 21.15 |
| *Pristimantis citriogaster* | 45.2 | 20 | 30.8 |
| *Pristimantis colonensis* | 18.65 | 6.75 | 10.36 |
| *Pristimantis condor* | 55.5 | 20.7 | 36 |
| *Pristimantis conspicillatus* | 49 | 18.5 | 29 |
| *Pristimantis cosnipatae* | 25.1 | 9.3 | 13.6 |
| *Pristimantis croceoinguinis* | 20.2 | 7.35 | 11.03 |
| *Pristimantis cruciocularis* | 20.3 | 7.6 | 10.5 |
| *Pristimantis danae* | 34 | 12.8 | 19.6 |
| *Pristimantis delius* | 30.9 | 12.3 | 16.8 |
| *Pristimantis dendrobatoides* | 29.5 | 10.2 | 23 |
| *Pristimantis diadematus* | 39.8 | 15.36 | 19.73 |
| *Pristimantis divnae* | 23.1 | 8.6 | 11.55 |
| *Pristimantis dundeei* | 23.6 | 7.9 | 13.3 |
| *Pristimantis epacrus* | 39.2 | 14.78 | 19.38 |
| *Pristimantis eurydactylus* | 34.7 | 13.1 | 17 |
| *Pristimantis exoristus* | 23.4 | 9 | 12.5 |
| *Pristimantis fenestratus* | 41.5 | 14.25 | 22.43 |
| *Pristimantis frater* | 21.5 | 8 | 10.5 |
| *Pristimantis galdi* | 21.4 | 8 | 13.2 |
| *Pristimantis gutturalis* | 30.4 | 9.9 | 17.3 |
| *Pristimantis imitatrix* | 15.8 | 5.6 | 8.4 |
| *Pristimantis infraguttatus* | 22.9 | 8.9 | 10.7 |
| *Pristimantis inguinalis* | 27 | 10.31 | 14.14 |
| *Pristimantis inusitatus* | 24 | 9.5 | 12.2 |
| *Pristimantis jester* | 21.2 | 8.4 | 11.86 |
| *Pristimantis kichwarum* | 20.2 | 7.6 | 12.3 |
| *Pristimantis lacrimosus* | 22.5 | 8.65 | 12.39 |
| *Pristimantis lanthanites* | 36.4 | 13.4 | 21.4 |
| *Pristimantis librarius* | 24.9 | 9.1 | 12.7 |
| *Pristimantis lindae* | 39.2 | 15.3 | 19.2 |
| *Pristimantis lirellus* | 21.5 | 8.2 | 11.8 |
| *Pristimantis luscombei* | 26.1 | 10.3 | 13.7 |
| *Pristimantis lythrodes* | 16.8 | 5.9 | 9.2 |
| *Pristimantis malkini* | 33.8 | 12.5 | 18.3 |
| *Pristimantis marmoratus* | 22 | 8 | 11 |
| *Pristimantis martiae* | 22.2 | 7.8 | 10.3 |
| *Pristimantis memorans* | 31.5 | 12.5 | 17.8 |
| *Pristimantis mendax* | 23 | 9.3 | 12.9 |
| *Pristimantis minutulus* | 17 | 6 | 9.6 |
| *Pristimantis muscosus* | 46.1 | 18.3 | 23.1 |
| *Pristimantis nebulosus* | 26.3 | 10.63 | 14.28 |
| *Pristimantis nephophilus* | 29.7 | 11.6 | 15.9 |
| *Pristimantis nigrogriseus* | 26.15 | 10.36 | 14.02 |
| *Pristimantis ockendeni* | 26.5 | 10.07 | 14.07 |
| *Pristimantis olivaceus* | 19.8 | 7.2 | 9.5 |
| *Pristimantis orcus* | 34 | 13 | 15.7 |
| *Pristimantis orphnolaimus* | 33.4 | 13.7 | 16.1 |
| *Pristimantis paulodutrai* | 35 | 10 | 19.75 |
| *Pristimantis paululus* | 19.4 | 7.1 | 9.6 |
| *Pristimantis pecki* | 17.8 | 7.2 | 9.3 |
| *Pristimantis percnopterus* | 25.9 | 10.2 | 14.1 |
| *Pristimantis peruvianus* | 41.5 | 17.9 | 23.28 |
| *Pristimantis petersi* | 19.9 | 7.4 | 10.2 |
| *Pristimantis platydactylus* | 30 | 11 | 14.2 |
| *Pristimantis pruinatus* | 26.8 | 12.9 | 12.9 |
| *Pristimantis pseudoacuminatus* | 20 | 6.76 | 13.56 |
| *Pristimantis pulvinatus* | 26.1 | 9.8 | 14.1 |
| *Pristimantis quaquaversus* | 26.1 | 10.4 | 13.8 |
| *Pristimantis ramagii* | 22 | 7.63 | 13.06 |
| *Pristimantis rhabdolaemus* | 29.7 | 10.2 | 17.3 |
| *Pristimantis rufioculis* | 20.6 | 7.7 | 11.5 |
| *Pristimantis saltissimus* | 22.5 | 8.6 | 12.42 |
| *Pristimantis savagei* | 21.2 | 7.8 | 11.5 |
| *Pristimantis skydmainos* | 30.5 | 12.5 | 18.3 |
| *Pristimantis tantanti* | 20.75 | 7 | 11.1 |
| *Pristimantis toftae* | 21.1 | 7.3 | 12.4 |
| *Pristimantis variabilis* | 24.35 | 8.21 | 13.02 |
| *Pristimantis ventrimarmoratus* | 36.9 | 14.05 | 18.05 |
| *Pristimantis versicolor* | 22.2 | 8.7 | 12.4 |
| *Pristimantis vilarsi* | 38.11 | 13.33 | 20.2 |
| *Pristimantis vilcabambae* | 22.1 | 8.1 | 10.4 |
| *Pristimantis vinhai* | 25 | 9 | 12.1 |
| *Pristimantis w.nigrum* | 59 | 20.5 | 38 |
| *Pristimantis waoranii* | 29.76 | 13.57 | 15.77 |
| *Pristimantis yaviensis* | 28.1 | 12 | 14.4 |
| *Pristimantis zeuctotylus* | 25.4 | 10 | 14.2 |
| *Pristimantis zimmermanae* | 19.1 | 6.9 | 9.5 |
| *Pristimantis zoilae* | 28.39 | 10.98 | 14.78 |
| *Proceratophrys appendiculata* | 48.5 | 25.8 | 21.6 |
| *Proceratophrys avelinoi* | 33.58 | 15.33 | 11.25 |
| *Proceratophrys bigibbosa* | 53.4 | 23.8 | 16.8 |
| *Proceratophrys boiei* | 62.1 | 31.4 | 23.9 |
| *Proceratophrys brauni* | 39.8 | 19.2 | 14.1 |
| *Proceratophrys concavitympanum* | 54 | 24.97 | 19.4 |
| *Proceratophrys cristiceps* | 44.7 | 19.7 | 16.1 |
| *Proceratophrys cururu* | 50.45 | 23.2 | 19.15 |
| *Proceratophrys goyana* | 59.3 | 26.3 | 21.9 |
| *Proceratophrys laticeps* | 71.5 | 42.5 | 24.5 |
| *Proceratophrys melanopogon* | 52.2 | 26.3 | 19 |
| *Proceratophrys moehringi* | 60 | 33 | 25.2 |
| *Proceratophrys moratoi* | 35.7 | 15 | 12.5 |
| *Proceratophrys palustris* | 29.83 | 14.95 | 10.4 |
| *Proceratophrys paviotii* | 51 | 23.6 | 18.4 |
| *Proceratophrys phyllostomus* | 55.4 | 27.6 | 19 |
| *Proceratophrys schirchi* | 46 | 17 | 20 |
| *Proceratophrys subguttata* | 47.3 | 26.7 | 17.8 |
| *Proceratophrys vielliardi* | 40.3 | 18 | 15.4 |
| *Pseudis bolbodactyla* | 43.1 | 16.2 | 24 |
| *Pseudis cardosoi* | 42.2 | 14.87 | 21.42 |
| *Pseudis fusca* | 43.45 | 16.65 | 25.4 |
| *Pseudis minuta* | 45.8 | 15.78 | 25.23 |
| *Pseudis paradoxa* | 56 | 19 | 32 |
| *Pseudis platensis* | 50 | 16 | 25 |
| *Pseudis tocantins* | 36.8 | 15.4 | 22.6 |
| *Pseudopaludicola boliviana* | 13.4 | 4.6 | 7.1 |
| *Pseudopaludicola canga* | 18.2 | 6.1 | 8.3 |
| *Pseudopaludicola ceratophyes* | 13 | 4.5 | 7.9 |
| *Pseudopaludicola falcipes* | 14.95 | 5.2 | 7.25 |
| *Pseudopaludicola llanera* | 15.9 | 5.2 | 7.4 |
| *Pseudopaludicola mineira* | 17.4 | 6.3 | 8 |
| *Pseudopaludicola mystacalis* | 18.9 | 6.63 | 10 |
| *Pseudopaludicola saltica* | 20.2 | 7.29 | 14 |
| *Pseudopaludicola ternetzi* | 19.9 | 6.7 | 10.25 |
| *Psychrophrynella usurpator* | 24.1 | 7.4 | 10 |
| *Ranitomeya amazonica* | 17.5 | 5.59 | 8.17 |
| *Ranitomeya benedicta* | 17.2 | 5.5 | 8.3 |
| *Ranitomeya fantastica* | 20 | 6.56 | 9.07 |
| *Ranitomeya flavovittata* | 18.1 | 6.01 | 7.31 |
| *Ranitomeya imitator* | 19 | 6 | 7 |
| *Ranitomeya reticulata* | 17 | 5.61 | 7.78 |
| *Ranitomeya sirensis* | 16.8 | 5.4 | 6.4 |
| *Ranitomeya summersi* | 19.5 | 6.3 | 8.5 |
| *Ranitomeya uakarii* | 15.39 | 5.08 | 7 |
| *Ranitomeya vanzolinii* | 17.5 | 6 | 7.3 |
| *Ranitomeya variabilis* | 26.8 | 9.03 | 14.1 |
| *Ranitomeya ventrimaculata* | 16 | 5 | 7 |
| *Rhaebo glaberrimus* | 66.4 | 22.3 | 26.2 |
| *Rhaebo guttatus* | 174.3 | 63.6 | 65.7 |
| *Rhaebo nasicus* | 66.5 | 24.82 | 32.59 |
| *Rhinella abei* | 81.9 | 29.1 | 17.1 |
| *Rhinella achavali* | 119 | 43.9 | 29.31 |
| *Rhinella acutirostris* | 57 | 19.84 | 20.92 |
| *Rhinella arenarum* | 66.03 | 24.7 | 21.6 |
| *Rhinella bergi* | 49.93 | 16.62 | 16.39 |
| *Rhinella castaneotica* | 39.5 | 13 | 14 |
| *Rhinella ceratophrys* | 93.9 | 31.1 | 44.8 |
| *Rhinella cerradensis* | 114.5 | 41.4 | 41.6 |
| *Rhinella cristinae* | 55.1 | 19.5 | 22.1 |
| *Rhinella crucifer* | 81.63 | 30.98 | 34.72 |
| *Rhinella dapsilis* | 77 | 27 | 31 |
| *Rhinella dorbignyi* | 55.58 | 19.15 | 16.23 |
| *Rhinella fernandezae* | 57.01 | 19.03 | 17.7 |
| *Rhinella festae* | 37 | 13.04 | 11.16 |
| *Rhinella fissipes* | 38.4 | 12 | 11.5 |
| *Rhinella granulosa* | 52.89 | 16.99 | 18.4 |
| *Rhinella henseli* | 58.27 | 19.87 | 26.69 |
| *Rhinella hoogmoedi* | 47.9 | 17.7 | 19.3 |
| *Rhinella humboldti* | 41 | 15 | 14.5 |
| *Rhinella icterica* | 137.3 | 53.55 | 49.43 |
| *Rhinella inca* | 39 | 14 | 20 |
| *Rhinella iserni* | 38.75 | 14 | 14.36 |
| *Rhinella jimi* | 133.8 | 48.63 | 45.37 |
| *Rhinella lescurei* | 43.7 | 15.95 | 18.45 |
| *Rhinella magnussoni* | 45.9 | 14.8 | 16.3 |
| *Rhinella manu* | 38.18 | 11.84 | 13.86 |
| *Rhinella margaritifera* | 68.9 | 25.88 | 29.36 |
| *Rhinella marina* | 97.5 | 36 | 38 |
| *Rhinella martyi* | 66.5 | 28 | 27.42 |
| *Rhinella nesiotes* | 29 | 11.7 | 11.2 |
| *Rhinella ocellata* | 59 | 20 | 19 |
| *Rhinella ornata* | 63.57 | 22.79 | 29.21 |
| *Rhinella poeppigii* | 123.78 | 45.55 | 45.67 |
| *Rhinella proboscidea* | 50.5 | 18.04 | 19.36 |
| *Rhinella pygmaea* | 40.76 | 13.84 | 12.46 |
| *Rhinella roqueana* | 74.5 | 28.06 | 31.06 |
| *Rhinella rubescens* | 120 | 45.53 | 40.58 |
| *Rhinella schneideri* | 250 | 95.32 | 104.07 |
| *Rhinella scitula* | 48 | 16.9 | 18.1 |
| *Rhinella spinulosa* | 80 | 30 | 30 |
| *Rhinella stanlaii* | 58.3 | 21.05 | 21.95 |
| *Rhinella veraguensis* | 55 | 19.21 | 20 |
| *Rhinella veredas* | 101.9 | 34.2 | 21.9 |
| *Rulyrana flavopunctata* | 24.9 | 9.01 | 14.31 |
| *Rulyrana mcdiarmidi* | 29.03 | 10.9 | 17.06 |
| *Rulyrana saxiscandens* | 22 | 7.7 | 12.9 |
| *Rulyrana spiculata* | 26.1 | 9.49 | 15.5 |
| *Scarthyla goinorum* | 20 | 6 | 12.18 |
| *Scinax acuminatus* | 45 | 16.14 | 21.06 |
| *Scinax alter* | 28.3 | 9.3 | 14.1 |
| *Scinax auratus* | 22.8 | 7.4 | 13 |
| *Scinax baumgardneri* | 29 | 9 | 13.5 |
| *Scinax blairi* | 32.3 | 10.17 | 14.9 |
| *Scinax boesemani* | 31.4 | 11 | 15.5 |
| *Scinax cabralensis* | 23.7 | 8.5 | 11.9 |
| *Scinax caldarum* | 28 | 8.82 | 16 |
| *Scinax camposseabrai* | 31.2 | 7.9 | 13.1 |
| *Scinax cardosoi* | 19.6 | 6.1 | 10 |
| *Scinax chiquitanus* | 33.5 | 10 | 17.2 |
| *Scinax constrictus* | 31.93 | 10.67 | 17.64 |
| *Scinax cretatus* | 30.7 | 10.5 | 15.8 |
| *Scinax crospedospilus* | 31 | 9.92 | 15.81 |
| *Scinax cruentomma* | 28 | 8.9 | 13.4 |
| *Scinax curicica* | 30.4 | 9.8 | 14.7 |
| *Scinax cuspidatus* | 27 | 9 | 15.5 |
| *Scinax danae* | 26.2 | 8.91 | 13.1 |
| *Scinax duartei* | 37 | 11 | 18 |
| *Scinax eurydice* | 42 | 14 | 23 |
| *Scinax exiguus* | 19.8 | 6.14 | 9.31 |
| *Scinax funereus* | 35 | 11.4 | 17.9 |
| *Scinax fuscomarginatus* | 20.4 | 5.7 | 9.9 |
| *Scinax fuscovarius* | 42.5 | 12.5 | 22 |
| *Scinax garbei* | 41.3 | 12.8 | 23.9 |
| *Scinax granulatus* | 34 | 12 | 18 |
| *Scinax hayii* | 48 | 15.84 | 24.96 |
| *Scinax ictericus* | 32 | 10.3 | 16.2 |
| *Scinax iquitorum* | 38.5 | 12.7 | 18.9 |
| *Scinax jolyi* | 124.19 | 49.05 | 61.99 |
| *Scinax karenanneae* | 30.5 | 10.3 | 15.8 |
| *Scinax kennedyi* | 37.3 | 11.5 | 20.3 |
| *Scinax lindsayi* | 26.3 | 8.4 | 13.3 |
| *Scinax maracaya* | 27.58 | 8.75 | 15.25 |
| *Scinax nasicus* | 31 | 10.02 | 15.55 |
| *Scinax nebulosus* | 37.5 | 12.84 | 19.81 |
| *Scinax oreites* | 36.5 | 11.4 | 18.7 |
| *Scinax pachycrus* | 33 | 10 | 19 |
| *Scinax pedromedinae* | 29.4 | 9.3 | 15.8 |
| *Scinax perereca* | 42.2 | 13.2 | 20 |
| *Scinax proboscideus* | 37.4 | 12.81 | 20.26 |
| *Scinax rostratus* | 42 | 14.1 | 24.2 |
| *Scinax ruber* | 40.3 | 12.7 | 20.5 |
| *Scinax similis* | 35 | 11 | 16.5 |
| *Scinax squalirostris* | 27 | 9 | 14 |
| *Scinax tigrinus* | 29.6 | 9.4 | 15.9 |
| *Scinax wandae* | 24.9 | 7.7 | 12 |
| *Scinax x.signatus* | 42.5 | 13.6 | 21.25 |
| *Scythrophrys sawayae* | 17.5 | 6 | 9 |
| *Sphaenorhynchus bromelicola* | 28 | 9 | 14.05 |
| *Sphaenorhynchus caramaschii* | 27.67 | 8.38 | 13 |
| *Sphaenorhynchus carneus* | 15.8 | 5.5 | 8.62 |
| *Sphaenorhynchus dorisae* | 27.5 | 9.1 | 12.34 |
| *Sphaenorhynchus lacteus* | 42.6 | 14.81 | 19.36 |
| *Sphaenorhynchus mirim* | 17.2 | 5.2 | 7.2 |
| *Sphaenorhynchus orophilus* | 30.3 | 8.79 | 13.33 |
| *Sphaenorhynchus palustris* | 34 | 11 | 16.37 |
| *Sphaenorhynchus pauloalvini* | 20 | 6.4 | 8.88 |
| *Sphaenorhynchus planicola* | 24 | 7 | 12 |
| *Sphaenorhynchus prasinus* | 31 | 9.8 | 13.94 |
| *Sphaenorhynchus surdus* | 25.78 | 7.75 | 12.53 |
| *Stefania ackawaio* | 46.48 | 18.15 | 31.14 |
| *Stefania ayangannae* | 44.67 | 18 | 30.82 |
| *Stefania breweri* | 49.6 | 18.5 | 32.5 |
| *Stefania coxi* | 67.23 | 27.16 | 43.03 |
| *Stefania evansi* | 63.9 | 26.89 | 39.81 |
| *Stefania ginesi* | 55 | 23 | 33 |
| *Stefania goini* | 92.2 | 40.5 | 60 |
| *Stefania marahuaquensis* | 27 | 10 | 15.9 |
| *Stefania oculosa* | 55.3 | 22.6 | 33.1 |
| *Stefania percristata* | 39.6 | 15 | 23.9 |
| *Stefania riae* | 59 | 21.77 | 34.57 |
| *Stefania roraimae* | 46 | 17.63 | 28.88 |
| *Stefania satelles* | 56.6 | 21.2 | 29.5 |
| *Stefania scalae* | 49.8 | 20.71 | 31.87 |
| *Stefania schuberti* | 76 | 33.8 | 42 |
| *Stefania tamacuarina* | 50 | 20 | 30 |
| *Stefania woodleyi* | 60 | 21.5 | 34 |
| *Stereocyclops histrio* | 35.9 | 13.5 | 13.7 |
| *Stereocyclops incrassatus* | 41 | 15.5 | 17.5 |
| *Stereocyclops parkeri* | 37.15 | 13.01 | 14.82 |
| *Strabomantis cornutus* | 56.5 | 27 | 29 |
| *Strabomantis sulcatus* | 42 | 18.98 | 21.64 |
| *Synapturanus mirandaribeiroi* | 32.9 | 7.81 | 10.94 |
| *Synapturanus rabus* | 19 | 4.8 | 6.7 |
| *Synapturanus salseri* | 25.1 | 6.9 | 10.5 |
| *Telmatobius hintoni* | 80.6 | 35.66 | 36.59 |
| *Telmatobius macrostomus* | 65.03 | 29.47 | 29.39 |
| *Tepuihyla edelcae* | 34.5 | 11.6 | 17.8 |
| *Tepuihyla exophthalma* | 32.7 | 12 | 17.3 |
| *Tepuihyla rodriguezi* | 37 | 12.14 | 18.8 |
| *Tepuihyla tuberculosa* | 85.7 | 29.2 | 45.6 |
| *Teratohyla adenocheira* | 22.4 | 9.4 | 12.4 |
| *Teratohyla amelie* | 18.2 | 6.6 | 11.1 |
| *Teratohyla midas* | 22.7 | 8.17 | 13.1 |
| *Thoropa lutzi* | 27.5 | 10.5 | 15.5 |
| *Thoropa megatympanum* | 43.2 | 18.7 | 21.2 |
| *Thoropa miliaris* | 71.1 | 28.44 | 39.11 |
| *Thoropa petropolitana* | 21 | 7.5 | 12 |
| *Thoropa saxatilis* | 57.5 | 23.9 | 33.9 |
| *Trachycephalus atlas* | 98 | 30 | 27.13 |
| *Trachycephalus coriaceus* | 57.5 | 18.63 | 29.48 |
| *Trachycephalus dibernardoi* | 77.58 | 25.38 | 39.47 |
| *Trachycephalus hadroceps* | 53.9 | 19.6 | 25.8 |
| *Trachycephalus imitatrix* | 53 | 16 | 26 |
| *Trachycephalus lepidus* | 58.7 | 20.1 | 33.1 |
| *Trachycephalus mesophaeus* | 67 | 21 | 32 |
| *Trachycephalus nigromaculatus* | 86 | 26 | 36 |
| *Trachycephalus resinifictrix* | 80 | 25 | 37 |
| *Trachycephalus typhonius* | 88.6 | 29.24 | 38.63 |
| *Vitreorana eurygnatha* | 22 | 8 | 11 |
| *Vitreorana gorzulae* | 21.4 | 8.7 | 12.9 |
| *Vitreorana parvula* | 17 | 6.06 | 9.11 |
| *Vitreorana ritae* | 19 | 7.5 | 11.5 |
| *Vitreorana uranoscopa* | 25 | 9 | 14 |
| *Xenohyla eugenioi* | 42.5 | 13.25 | 16.85 |
| *Xenohyla truncata* | 39.78 | 11.83 | 15.42 |
| *Yunganastes mercedesae* | 44.8 | 16.95 | 31.2 |
| *Zachaenus carvalhoi* | 35 | 14 | 16 |
| *Zachaenus parvulus* | 20.5 | 9.5 | 9 |

**Table S3**. Species traits selected and included in the functional analises per 0.5 x 0.5 grid for the Amazon, the Cerrado and the Atlantic Rainforest. We provided the biological interpretation of each trait as well as its ecological function for a clearer interpretation of the reason why to include each one of them.

| **Type of development** | **Trait** | **Biological interpretation** | **Ecological function** | **Type of metric** |
| --- | --- | --- | --- | --- |
| Adult | Snout vent length | Higher values represent a greater dispersion ability | Survival (Cabrera-Guzmán et al. 2013), Dispersal ability (Jenkins et al. 2007) | Continuous |
|  | Head width | Small values reflect more specific prey consumption | Diet-Prey consuption (Duellman & Trueb 1994), nutrient cycling (Cortéz-Gomez et al. 2016) | Continuous |
|  | Tibia length | Higher values represent a greater dispersion ability | Dispersal ability (Cortéz-Gomez et al. 2016) | Continuous |
|  | Reproductive mode | Reflect the type of reproduction | Anuran development (Duellman & Trueb 1994; Haddad & Prado 2005) | Categorical |
| Tadpole | Tadpoles type of development | *Exotrophic*: environmental role in nutrient cycling *Endotrophic*: absence of environmental nutrient cycling | Nutrient cycling (Whiles et al. 2006; Cortéz-Gomez et al. 2015, 2016) | Categorical |

**Supporting Information: Material S3. Trait imputation**

Whenever continuous trait data were missing from a set of species, we used Phylopars (Bruggeman *et al.*, 2009) to perform trait imputation. Imputation of missing data has been shown to reduce error and improve estimation in macroecological and evolutionary studies (Penone *et al.*, 2014). To that end, Phylopars perform imputation based on the phylogenetic relationship among species, which has been demonstrated to improve estimation of missing trait values (e.g., Guénard *et al.*, 2013; Penone *et al.*, 2014; Swenson, 2014). For imputation, we represented the role of phylogeny by applying a Brownian motion evolutionary model. Brownian motion assumes an evolutionary model of constant change, with variance accumulation in species traits being proportional to the time of lineage splitting (Revell *et al.*, 2008). As showed by Penone *et al.* (2014), Phylopars has superior performance compared to other methods of trait imputation. Because it is impossible to apply the imputation analysis for species missing all the trait data, we excluded six species before performing the imputation. So, in the end, the computation of functional diversity was done for 1090 species instead of 1096. The imputation of missing data was performed in R version 3.4.1, package Rphylopars (function *phylopars*, Goolsby *et al.*, 2017). Approximately 10% of the continuous trait data, and 5% of the categorical trait data, were estimated through imputation (see imputed trait table in Table S4).

To be sure that the imputation of the missing data was not changing the evolutionary pattern of the phylogenetic tree, we tested for phylogenetic signal in the imputed and non-imputed continuous trait dataset by estimating the K statistic of Blomberg *et al.* (2003). Blomberg’s K estimates the phylogenetic signal relatively to the number of evolutionary trait changes expected under Brownian motion (Blomberg *et al.*, 2003). In this approach, K < 1 indicates small phylogenetic signal in the data (i.e., closely-related species are less similar than expected by Brownian motion), K = 1 indicates that the trait evolution is happening according to the expected by Brownian motion, and K > 1 means high phylogenetic signal present in the data (i.e., closer species share more trait similarities than expected by Brownian motion). In the case of the categorical trait data, phylogenetic signal was tested with the method of Maddison & Slatkin, 1991, where the tree is fixed, but traits are reshuffled randomly. As in the case of Blomberg's K for continuous trait data, this method randomizes tree tips (taxa), and then compares the minimum number of character state changes (assuming maximum parsimony), with those under a null model, to estimate the minimum number of character-state transitions that account for the observed character distribution on the phylogeny. A significant phylogenetic signal is inferred when the randomized median is greater than the observed transition rates (Maddison & Slatkin, 1991). To estimate phylogenetic signal, we used 1,000 randomizations.

The strength of the phylogenetic signal emergent in the continuous data varied among domains, as shown by the values of Blomberg’s K (ranging from 0.048 to 0.062 in the Atlantic Rainforest, from 0.234 to 0.360 in the Cerrado, and from 0.3 to 0.394 in the Amazon; Table S3). For the continuous traits, there was less phylogenetic signal than expected under a Brownian Motion model. However, the opposite pattern is observed in the categorical trait data: all categorical traits exhibited significant phylogenetic signal (Table S4).

Table S4. Phylogenetic signal of continuous imputed traits across the three domains. Phylogenetic signal was quantified using Blomberg’s *K* statistics (*K*), with statistical significance assessed via phylogenetically independent contrasts (PIC; P-value and Z-score). Values of *K* < 1 indicate weaker phylogenetic signal than expected under Brownian motion, *K* = 1 indicates evolution consistent with Brownian motion, and *K* > 1 indicates stronger phylogenetic signal.

| Biome | Trait | *K* | PIC.variance.P | PIC.variance.Z |
| --- | --- | --- | --- | --- |
| Atlantic Forest | SVL | 0.062 | 0.001 | -1.0788357 |
|  | Head width | 0.048 | 0.016 | -0.9977475 |
|  | Tibia lenght | 0.055 | 0.004 | -1.1190054 |
| Cerrado | SVL | 0.345 | 0.001 | -4.053306 |
|  | Head width | 0.360 | 0.001 | -4.233683 |
|  | Tibia lenght | 0.234 | 0.001 | -3.549059 |
| Amazon | SVL | 0.394 | 0.001 | -5.278008 |
|  | Head width | 0.383 | 0.001 | -5.256102 |
|  | Tibia lenght | 0.300 | 0.001 | -5.054435 |

**Table S5**. Phylogenetic signal for imputed categorical data in the three biomes. The traits show phylogenetic signal when the observed number of changes in trait states is lower than the median null number of changes. *P* is the significance value.

| Biome | Trait | Nº of trait states | Observed nº of changes | Median null nº of changes | *P* |
| --- | --- | --- | --- | --- | --- |
| Atlantic Rainforest | Reproductive mode | 2 | 3 | 55 | 0 |
|  | Type of development | 3 | 14 | 80 | 0 |
| Cerrado | Reproductive mode | 2 | 3 | 15 | 0 |
|  | Type of development | 3 | 5 | 23 | 0 |
| Amazon | Reproductive mode | 2 | 13 | 138 | 0 |
|  | Type of development | 3 | 20 | 150 | 0 |

**Table S6**. Summary of Conditional Autoregressive (CAR) model results for the influence of the topographic heterogeneity (Relief aspect and slope), climatic stability, and current climate variables (precipitation of the driest quarter, bio17) in each biodiversity dimension. The beta coefficients and their significance are available in Table 1 in the main text.

| **Domain** | **Model** | **Full model** | **Predictors (*R*²)** | **AIC** | ***P*** |
| --- | --- | --- | --- | --- | --- |
| Atlantic Rainforest | TD ~ Relief + Climatic Stability + Climate | 0.908 | 0.305 | 3682.8 | < 0.001 |
| Cerrado | TD ~ Relief + Climatic Stability + Climate | 0.875 | 0.194 | 3794.3 | < 0.001 |
| Amazonia | TD ~ Relief + Climatic Stability + Climate | 0.960 | 0.209 | 11928.2 | < 0.001 |
| Atlantic Rainforest | FD ~ Relief + Climatic Stability + Climate | 0.852 | 0.382 | -3864.9 | < 0.001 |
| Cerrado | FD ~ Relief + Climatic Stability + Climate | 0.874 | 0.093 | -5712.2 | < 0.001 |
| Amazonia | FD ~ Relief + Climatic Stability + Climate | 0.897 | 0.283 | -15461.6 | < 0.001 |
| Atlantic Rainforest | PD ~ Relief + Climatic Stability + Climate | 0.812 | 0.112 | 625.7 | < 0.001 |
| Cerrado | PD ~ Relief + Climatic Stability + Climate | 0.765 | 0.05 | 389.1 | < 0.001 |
| Amazonia | PD ~ Relief + Climatic Stability + Climate | 0.877 | 0.300 | 2847.2 | < 0.001 |


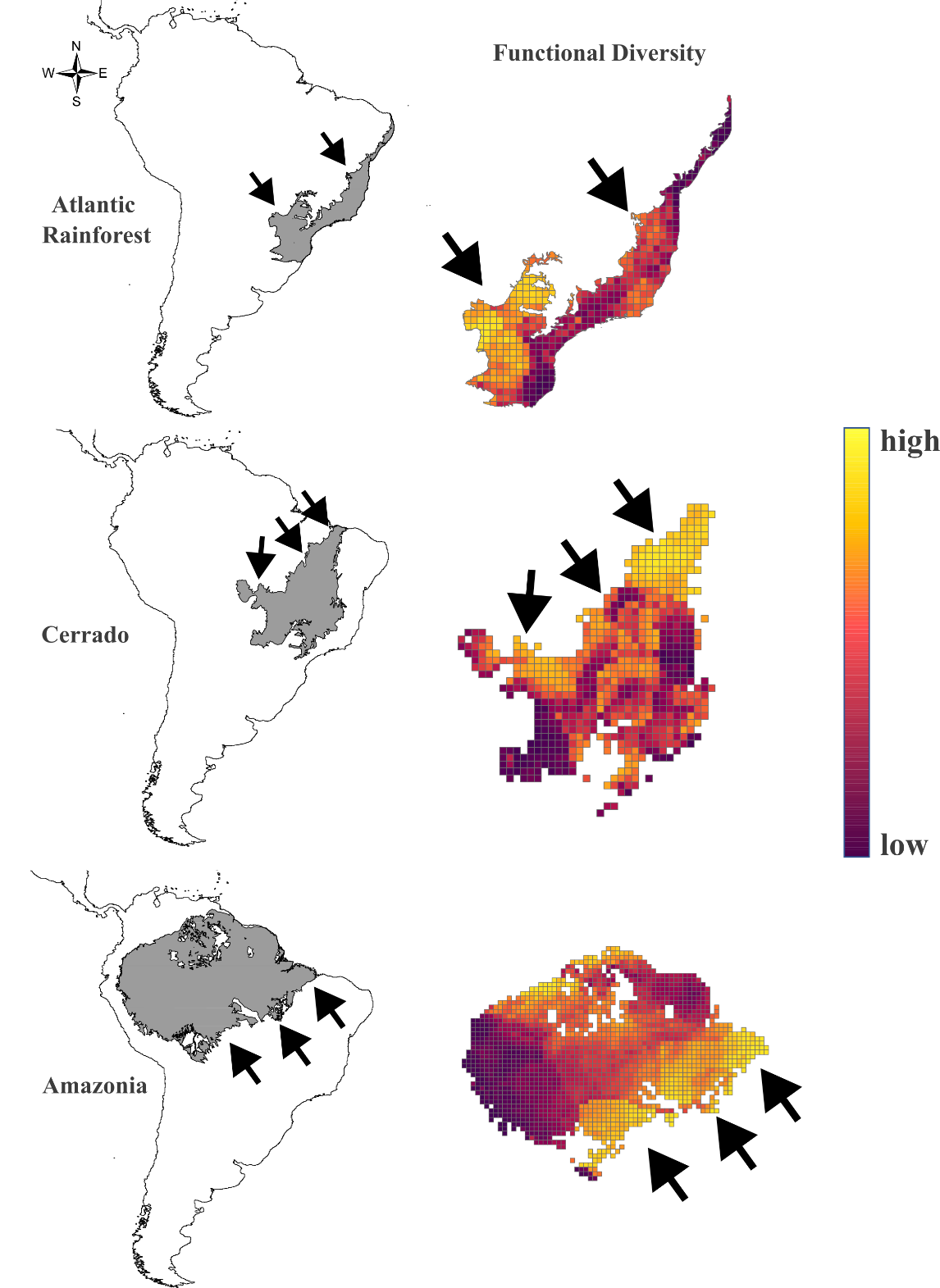


**Figure S5**. Spatial patterns of Functional Diversity (as in Figure 1, main text) emphasizing higher functional diversity (arrows pointing to yellowish grid cells) in adjacent, transitional zones (ecotones).

**
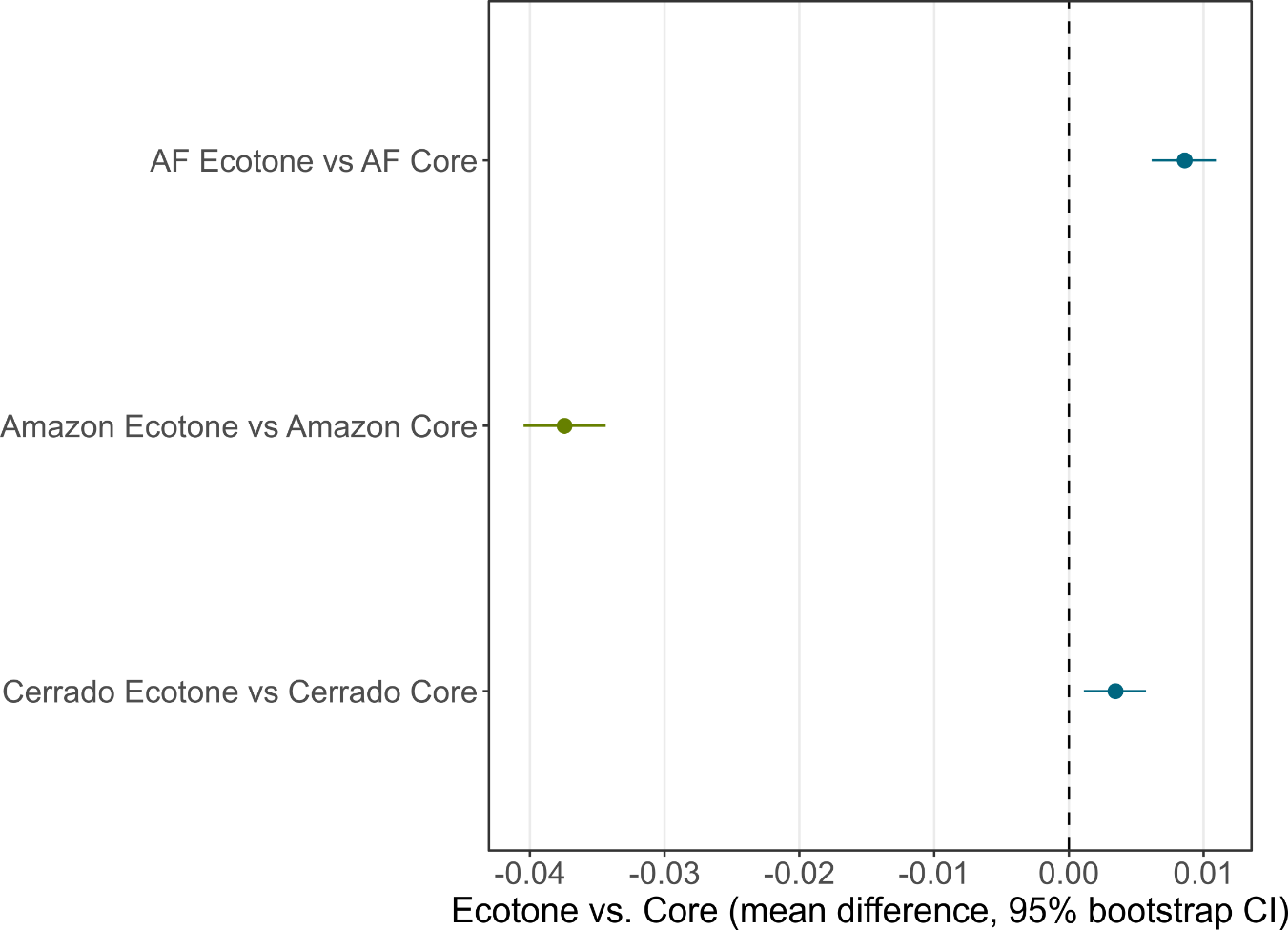
**

**Figure S6.** Differences in functional diversity (FD) between ecotone grid cells (cells adjacent to a neighboring biome) and core grid cells (interior cells of the same biome), shown as mean effects (points) with 95% bootstrap confidence intervals (lines); the dashed vertical line indicates no difference (0). Positive values (blue) indicate higher functional diversity in ecotone grid cells than in the biome core, whereas negative values (green) indicate higher functional diversity in the biome core than in ecotone grid cells.

**
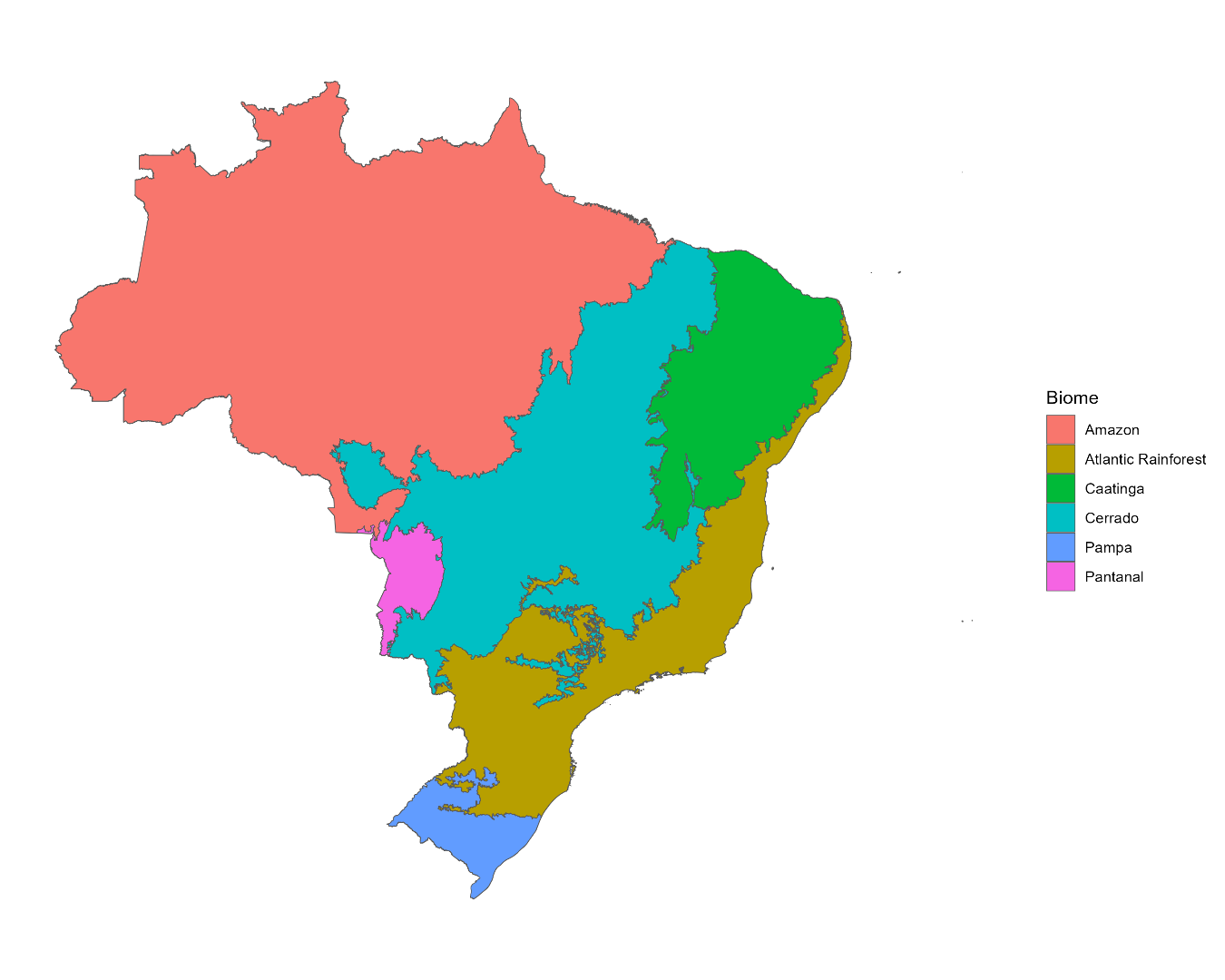
Figure S7**. Spatial distribution of terrestrial Brazilian biomes. Biome boundaries were obtained from official IBGE datasets using the geobr R package (Pereira and Gonçalves 2024, doi: 10.32614/CRAN.package.geobr.
